# Dendritic Spikes Amplify the Synaptic Signal to Enhance Detection of Motion in a Simulation of the Direction-Selective Ganglion Cell

**DOI:** 10.1371/journal.pcbi.1000899

**Published:** 2010-08-19

**Authors:** Michael J. Schachter, Nicholas Oesch, Robert G. Smith, W. Rowland Taylor

**Affiliations:** 1 Department of Neuroscience, University of Pennsylvania, Philadelphia, Pennsylvania, United States of America; 2 Casey Eye Institute, Department of Ophthalmology, Oregon Health & Science University, Portland, Oregon, United States of America; Université Paris Descartes, Centre National de la Recherche Scientifique, France

## Abstract

The On-Off direction-selective ganglion cell (DSGC) in mammalian retinas responds most strongly to a stimulus moving in a specific direction. The DSGC initiates spikes in its dendritic tree, which are thought to propagate to the soma with high probability. Both dendritic and somatic spikes in the DSGC display strong directional tuning, whereas somatic PSPs (postsynaptic potentials) are only weakly directional, indicating that spike generation includes marked enhancement of the directional signal. We used a realistic computational model based on anatomical and physiological measurements to determine the source of the enhancement. Our results indicate that the DSGC dendritic tree is partitioned into separate electrotonic regions, each summing its local excitatory and inhibitory synaptic inputs to initiate spikes. Within each local region the local spike threshold nonlinearly amplifies the preferred response over the null response on the basis of PSP amplitude. Using inhibitory conductances previously measured in DSGCs, the simulation results showed that inhibition is only sufficient to prevent spike initiation and cannot affect spike propagation. Therefore, inhibition will only act locally within the dendritic arbor. We identified the role of three mechanisms that generate directional selectivity (DS) in the local dendritic regions. First, a mechanism for DS intrinsic to the dendritic structure of the DSGC enhances DS on the null side of the cell's dendritic tree and weakens it on the preferred side. Second, spatially offset postsynaptic inhibition generates robust DS in the isolated dendritic tips but weak DS near the soma. Third, presynaptic DS is apparently necessary because it is more robust across the dendritic tree. The pre- and postsynaptic mechanisms together can overcome the local intrinsic DS. These local dendritic mechanisms can perform independent nonlinear computations to make a decision, and there could be analogous mechanisms within cortical circuitry.

## Author Summary

The On-Off direction-selective ganglion cell (DSGC) found in mammalian retinas generates a directional signal, responding most strongly to a stimulus moving in a specific direction. The DSGC initiates spikes in its dendritic tree which are thought to propagate to the soma and brain with high probability. Both dendritic and somatic spikes in the DSGC display strong directional tuning, whereas postsynaptic potentials (PSPs) recorded in the soma are only weakly directional, indicating that postsynaptic spike generation markedly enhances the directional signal. We constructed a realistic computational model to determine the source of the enhancement. Our results indicate that the DSGC dendritic tree is partitioned into separate computational regions. Within each region, the local spike threshold produces nonlinear amplification of the preferred response over the null response on the basis of PSP amplitude. The simulation results showed that inhibition acts locally within the dendritic arbor and will not stop dendritic spikes from propagating. We identified the role of three mechanisms that generate direction selectivity in the local dendritic regions, which suggests the origin of the previously described “non-direction-selective region,” and also suggests that the known DS in the synaptic inputs is apparently necessary for robust DS across the dendritic tree.

## Introduction

The On-Off direction-selective ganglion cell (DSGC) of the mammalian retina spikes vigorously to moving stimuli, but only weakly to stationary light spots. It responds most strongly over a limited range of stimulus directions, and the direction producing the maximal response is called the “preferred” direction, while a stimulus moving in the opposite direction, called the “null” direction, produces little or no response [1]. We refer to such directionally-tuned spike responses as “direction-selective”. On-Off DSGCs are sharply bistratified neurons that respond with a transient depolarization and burst of spikes at both the onset (“On” response) and termination (“Off” response) of a bright stimulus within the receptive field. Similarly the leading edge of a bright bar crossing the receptive field will produce a transient On-response, and, if the bar is wide relative to the dendritic extent and the speed low enough, the trailing-edge will produce a distinct, temporally separate Off-response. In their original description of the DSGC, Barlow and Levick [2] noted that direction-selective spike output was produced for stimuli that covered only a small fraction of the dendritic arbor. They proposed that the synaptic mechanism comprised “subunits” that were repeated in an array across the receptive field. In contrast to most ganglion cells, which initiate spikes in the axon initial segment, the DSGC initiates spikes in the dendritic tree [3]. The dendritic spikes are thought to propagate to the soma and initiate a somatic spike, similar to neurons in other regions of the brain where dendritic spiking is important for signal processing [4]. These observations suggest that some type of local dendritic processing could provide the basis for the proposed subunits.

**Figure 1:**
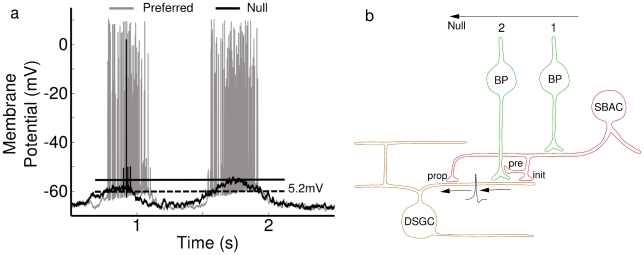
Direction-selective responses of the DSGC raise the question of how spikes are modulated by stimulus direction. (**a**) Direction-selective spike responses do not correlate with somatic PSP amplitudes. Current clamp responses from a DSGC soma during stimulation by a moving bright bar that advanced over its receptive field in the preferred (gray) and null (black) directions. The first burst of spikes occurred when the leading edge of the bar crossed the receptive field (On-response), and the second burst of spikes was produced by the trailing edge (Off-response). The solid horizontal line shows the peak somatic depolarization during the null-direction trailing edge response. The dashed line shows the threshold membrane potential for the first spike generated by the leading edge response, which was 5.2 mV more hyperpolarized than the peak of the null-direction PSP. Such responses preclude a single, common spike initiation zone in the soma or initial axon segment [3]. (**b**) Postulated mechanisms for modulation of spikes recorded in DSGC. Excitation from bipolar cells (BP) initiates a dendritic spike, which propagates to the soma and generates a somatic spike. Cholinergic excitation, from starburst amacrine cells (SBACs), is not shown here for simplicity. During Null motion, activation at position 1 generates inhibition that precedes the excitation activated at position 2. This GABAergic inhibition arises from asymmetrically connected SBACs and is critical for generating directional responses. Three possible mechanisms, not mutually exclusive, could produce direction-selective dendritic spikes: Postsynaptic inhibition on-the-path to the soma **(“prop”)** blocks propagation of spikes; Postsynaptic inhibition from SBACs **(“init”)** within a local dendritic region blocks spike initiation; Presynaptic inhibition of excitatory synaptic inputs **(“pre”)** to a local dendritic region suppresses spike initiation. A fourth mechanism not illustrated here is termed “intrinsic” and arises due to the asymmetry of each synaptic locus within the dendritic arbor, with respect to the whole dendritic arbor. This intrinsic morphological asymmetry produces local directional asymmetries in the PSP amplitude that may influence the size of local directional signals, as outlined below in the text.

Evidence for dendritic spiking in the DSGC was observed in low amplitude “spikelets”, which appear when somatic spiking is suppressed by local application of tetrodotoxin (TTX) to the soma, or by hyperpolarizing the soma [3]. Dendritic spikes are hypothesized to initiate somatic spikes with high probability because they are rarely seen under normal conditions. Further, both somatic and dendritic spiking responses are strongly tuned to preferred-direction stimuli, whereas the somatic graded potential shows relatively weak directional tuning (Figure 1a) [1]–[3]. This implies that the DSGC does not employ the mechanism used by most other ganglion cells for synaptic integration, where spikes initiated at the soma reflect the summation of synaptic inputs over the dendritic tree [5]. Instead it suggests that DSGC dendrites sum synaptic inputs and generate local spikes which then propagate to the soma, in the process amplifying the responses' directional selectivity.

In addition to dendritic spiking in the DSGC, other mechanisms are also important for generating its direction-selective response. GABAergic inhibition is essential, and presynaptic mechanisms render both excitatory and inhibitory synaptic inputs to the DSGC directionally-tuned [6], [7]. Both excitatory and inhibitory inputs vary in amplitude and relative timing as a function of direction. Further, postsynaptic integration of excitatory and inhibitory inputs has been hypothesized to contribute to DS signals [8]–[12]. Postsynaptic inhibition resulting from null direction movement could produce DS signals in two ways: it could block the propagation of dendritic spikes or it could block their initiation [2]–[4], [13] (Figure 1b).

However, the relative contributions of presynaptic and postsynaptic mechanisms to the DS spiking of the DSGC remains unclear. Initial theoretical studies suggested that postsynaptic mechanisms might suffice [14] and this received some experimental support [15]. However, more recently, presynaptic mechanisms have appeared to be the most significant [6], [7], [16], [17]. We wanted to revisit this issue to delineate the relative contributions of presynaptic and postsynaptic mechanisms in a calibrated model.

To investigate how dendritic processing of synaptic PSPs (postsynaptic potentials) could amplify DS, we constructed multi-compartment biophysical models of DSGCs, digitized from tracer-injected morphologies calibrated to physiological data obtained prior to tracer injection. We stimulated the models with moving light bars that activated synaptic inputs. The goal was to explore how morphology, voltage-gated channels, and synaptic inhibition affect the initiation and propagation of dendritic spikes, and to compare these with the known physiological properties. Our simulations show that sub-threshold PSPs from the distal dendritic regions of the On-Off DSGC are heavily attenuated by propagation to the soma, but that spikes initiated within local dendritic regions can propagate with high probability to the soma and back-propagate to the remainder of the dendritic tree. Therefore active amplification of DS appears to take place during spike initiation in the dendrites.

## Results

The responses of DSGCs are characterized by strong spiking in response to motion in the preferred direction, and little if any response to motion in the null direction (Figure 1). However, the responses in Figure 1 suggest that the slowly rising somatic membrane potential is not the main determinant of spike generation, because the peak of the trailing-edge PSP in the null-direction is ∼5 mV more depolarized than the apparent spike threshold for leading-edge motion in the preferred direction. In these bistratified neurons, the leading edge On-response and the trailing edge Off-response are mediated through inputs to the On-dendritic arbor and Off-dendritic arbor respectively. These two arbors are physically distinct, and directional signals are generated independently within each arbor [3],[18]. Figure 1 shows, consistent with previous data [3], that the spike-threshold depends on the dendritic source of the input. For the response illustrated, inputs to the On-dendritic arbor appear to have a lower threshold than for the Off-arbor. This is inconsistent with a simple integrate-and-fire model, and suggests initiation of spikes within the dendritic arbors of DSGCs [3].

Such striking results raised several questions and hypotheses: a) does the DSGC dendritic tree comprise local computational subunits that can support independent mechanisms for spike initiation or propagation; b) how do the propagation of dendritic PSPs and spikes differ; c) is directional tuning, reflected in somatic spiking, produced by selective spike initiation, or by selective dendritic spike propagation; d) can known inhibitory conductances in the DSGC support such putative selective tuning mechanisms; e) how accurate are voltage-clamp estimates of conductances in the DSGC? f) are the directional-differences in excitatory and inhibitory synaptic conductances the only determinant of spike directional selectivity, or are other mechanisms involved; g) do known or postulated presynaptic and postsynaptic mechanisms suffice to modulate spike directional selectivity, and what are their relative contributions; h) can dendritic computational subunits explain the lack of correlation between somatic PSPs and spikes?

### Distal dendrites comprise independent high-gain regions

**Figure 2:**
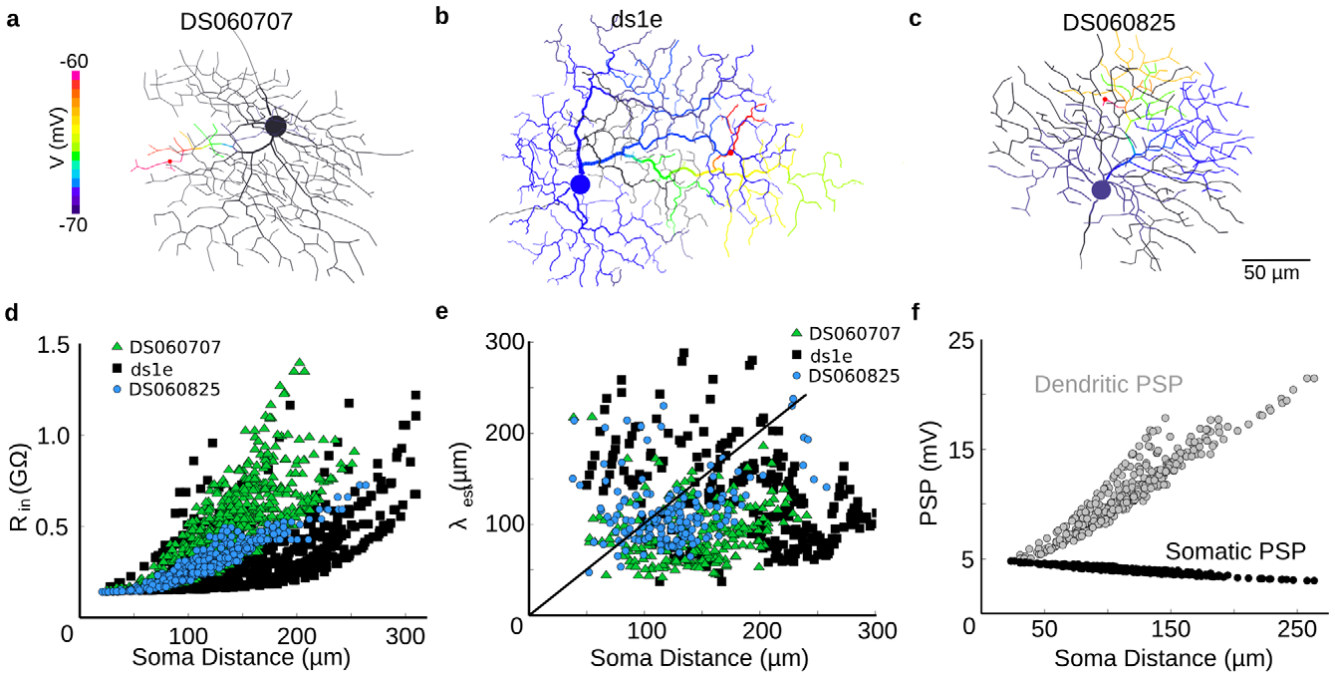
Dendrites of the DSGC are electrotonically isolated. (**a, b, c**) In a passive model without voltage-gated channels, a weak synaptic input (50pS, 100ms) excited a point in the dendritic tree, and the amplitude of the PSP spreading across the dendritic tree is shown in color, 50 ms after the onset of the stimulus. (**d**) Plot of input resistance (R_in_) vs. distance from soma for the morphologies shown above (green triangle = a, black square = b, blue circle = c). (**e**) Estimated local average space constant (see Methods) lambda_est_ versus distance from soma for the three morphologies. Symbols and colors same as in (d). The black line is unity (soma distance = lambda _est_). (**f**) Amplitude of subthreshold PSPs from single synapses across an active dendritic tree for the morphology shown in (c) at the soma (black circles) and in the dendrites (gray circles). Dendritic membrane and axial resistivities were uniform, with Rm = 40,000Ω·cm^2^ and Ri = 200Ω·cm (see Methods). For reference, these values produce lambda = 500 µm in a passive infinite cable of diameter 0.5µm. Lamba _est_ was smaller than this reference value because many dendrites in our model had a diameter less than 0.5um, and impedance mismatches and branch points between cables further contributed to substantial attenuation of voltage spread.

To explore whether the morphology of the DSGCs would provide for local dendritic processing, we measured the electrotonic properties. DSGC dendrites branch extensively, with higher-order dendrites tending to loop back towards the soma and many dendritic tips throughout the dendritic field [19], [20]. Dendritic diameter decreases with branching, ranging from 2–3µm for proximal dendrites to less than 0.5µm at terminal branches [21]. These morphological properties are typical of many neurons, especially retinal ganglion cells, and result in higher local input resistance and shorter electronic lengths as one moves away from the soma [22]–[25]. We mapped the input resistance for DSGCs in models that included all the voltage-gated channel types, and found, as in classical studies [22], that dendritic R_in_ increased with distance from soma, ranging in proximal dendrites from 150–200MΩ, to greater than 1GΩ for distal dendrites (Figure 2d). This implied that for a given excitatory synaptic conductance the distal dendrites generated larger PSPs than proximal dendrites.

Next we explored how dendritic morphology influenced the passive spread of PSPs within DSGC dendrites. The PSP from a single excitatory synapse (red spot, Figure 2a–c) produced strong local depolarization (magenta branches) that declined steeply with distance from the current-injection point [22]–[26]. We measured the degree of attenuation as a function of synaptic location by comparing the PSP amplitude in the dendrite with that at the soma (see Methods). Measurements of PSPs at single synapses at many points across the dendritic tree showed that dendritic PSP amplitude increased sharply with distance from soma, in line with the local input resistance values (Figure 2d). The corresponding somatic PSP amplitude was weakly dependent on synapse location, and declined gradually as the input was moved away from the soma (Figure 2f). This weak spatial dependence arises because the soma is centrally located, and, as evident from the relatively slow time-to-peak and decay time of the somatic PSPs, tends to reflect the overall depolarization reached after the charge injected into the dendrite has spread through the cell. Thus, under physiological conditions, at each point within the dendritic arbor, the synaptic depolarization will comprise a slower, spatially averaged component, generated by the total input to the cell, and a faster, higher-amplitude component generated by local inputs in the electrotonically isolated regions [23], [24].

We further quantified the electrotonic isolation by estimating the local space constant across the dendritic tree (see Methods), and found that the space constant of most dendritic loci was less than the distance to soma (Figure 2e), consistent with spatially localized PSPs. Overall the simulations suggest that the dendritic tree of the DSGC is composed of high-gain electrical subunits that can independently integrate synaptic input. This is true for both passive models and our calibrated active models, even when channel densities are perturbed from their “standard” values (see Methods). These high-gain subunits are proposed to generate the directional signals that drive the direction-selective dendritic spikes, which in turn enhance the directional tuning, as reported previously [3].

### Distal dendritic regions are highly excitable

**Figure 3 pcbi-1000899-g003:**
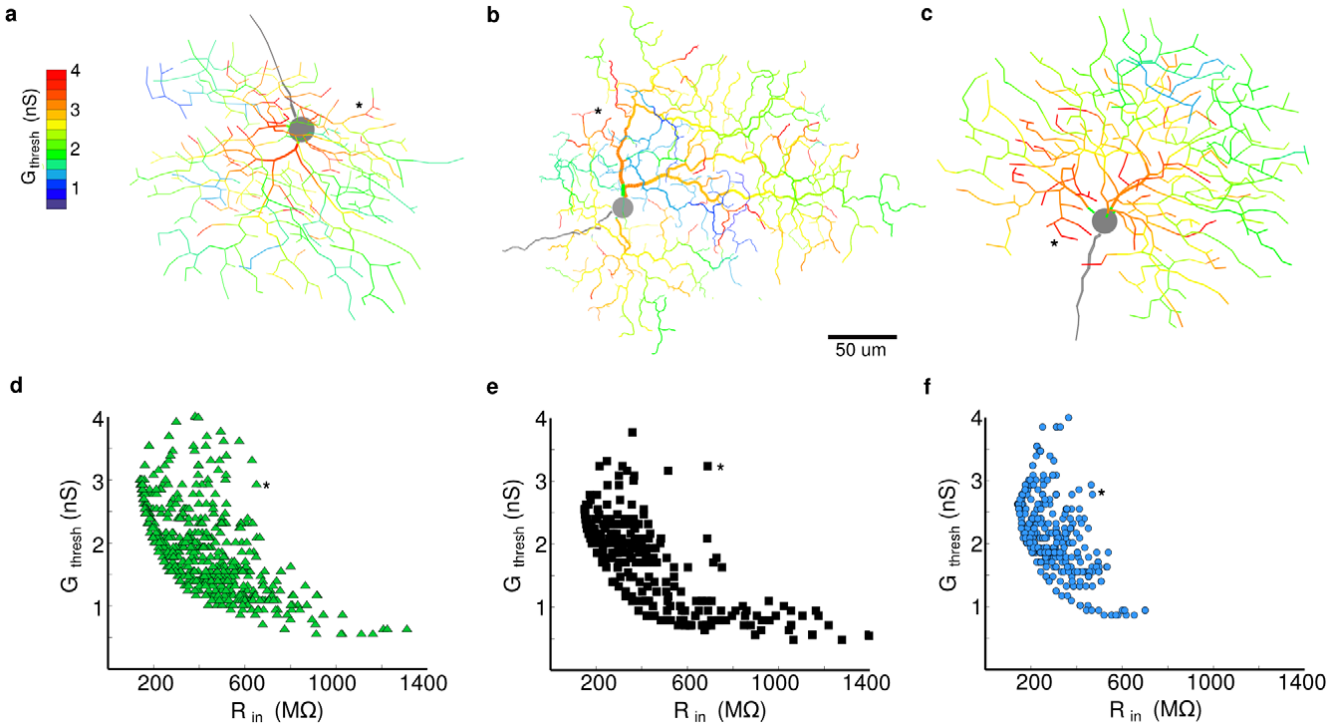
Peripheral dendritic tips are more excitable than proximal dendrites. (**a–c**) The minimum conductance (color) necessary for a single synapse to elicit a dendritic spike at points throughout each dendritic tree with uniform Na-channel density (“conductance threshold”, Gthresh, see Methods). Asterisks mark regions with medium Rin but high Gthresh (see Results). (**d–f**) Each morphology shows an inverse relationship between the conductance threshold and input resistance. Same symbols and colors as in Figure 2.

To measure the excitability of dendritic regions, we simulated dendritic spiking in models with uniformly high (g_Na1.6_ = 40 mS/cm^2^) dendritic Na^+^ channel densities. We activated a single synapse and measured G_thresh_, the “conductance threshold” for spiking, at locations sampled evenly and independently across the dendritic tree (see Methods, Figure 3). The locus of spike initiation was not always at the point of input but typically nearby, usually over an entire subregion (50–100 µm dia) within 1ms. Spikes did not initiate at the soma except for very proximal synaptic locations. Our first expectation was that R_in_ would be the predominant determinant of G_thresh_, i.e. G_thresh_ would be inversely proportional to R_in_, however the scatter of the points in Figs. 2d–f show that R_in_ is not the overriding factor. A small number of locations at intermediate distances from the soma had higher thresholds than would be predicted from R_in_ alone (asterisks in Figure 3a–c). These regions had few nearby distal branches with high R_in_ that could be charged up to produce a spike, and experienced significant axial current flow through a high-conductance pathway to the soma [24]. This reduced the current available to charge the local capacitance, causing a rate-of-rise insufficient to produce a spike, but a PSP amplitude high enough to inactivate Na^+^ channels (more depolarized than −50mV). We also found that the threshold was higher at some of the extreme dendritic tips, due to their higher axial resistance, which reduced their ability to excite more proximal dendrites. However, the majority of locations had bi-directional current paths with proximity to highly excitable terminal dendrites, and therefore had a low spike threshold. These effects implied that the spike threshold of a single dendritic location was dependent on the properties of the local dendritic region. Overall, the distal dendrites of the DSGC, which cover most of the dendritic field [19], [21], comprise electrotonically isolated local regions with high gain and low spike threshold, and these regions are capable of independently integrating synaptic input to generate a dendritic spike.

In models with dendritic initiation of spiking, we observed that when a dendritic spike reached the soma it invariably spread throughout the entire cell and obliterated any simultaneous dendritic spikes. In these models, when a dendritic region received excitatory input, the dendrites within the local region of 50–100 µm in extent depolarized toward spike threshold. When several such regions received simultaneous excitatory input, the one that reached spike threshold first generated a full-blown spike that propagated to the soma within 1–2 ms, and then back-propagated into the other dendrites within 1–2 ms rendering them refractory (see Video S1).

### Spike propagation fails for models with low Na-channel density

**Figure 4 pcbi-1000899-g004:**
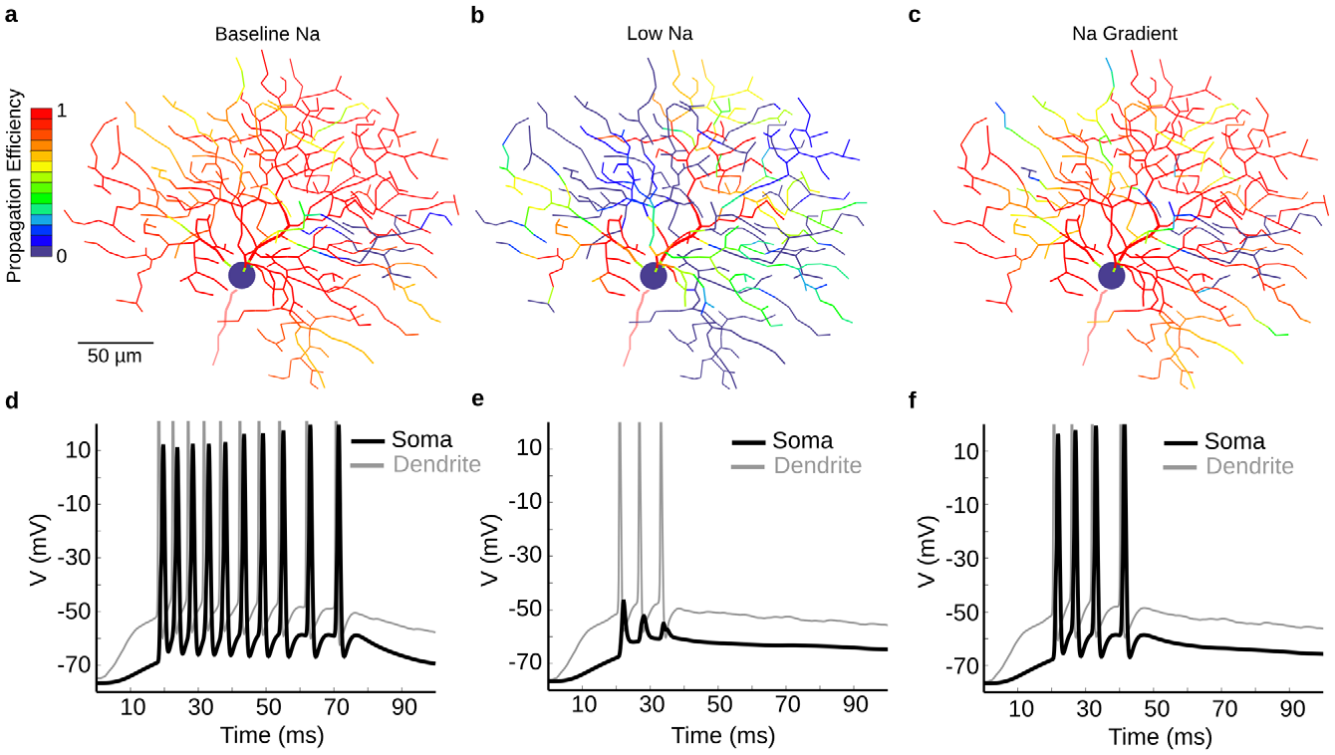
A gradient of Na-channels improves propagation in a model with low Na-channel density. (**a**) Propagation efficiency (number of somatic spikes/number of dendritic spikes) for spikes initiated at different dendritic locations in the calibrated model (gNa1.6 = 40mS/cm^2^). Most dendritic spikes propagated successfully to the soma. (**b**) Propagation efficiency of the low-Na model (gNa1.6 = 25mS/cm^2^) shows failure of propagation for most points. (**c**) Propagation efficiency for model with dendritic Na-channel gradient (proximal gNa1.6 = 45mS/cm^2^, distal gNa1.6 = 20mS/cm^2^) shows greatly improved propagation efficiency. (**d, e, f**) A “spot” of synaptic input 50µm in diameter stimulated a region in the dendritic tree, while voltage at soma (black) and dendrite (gray) was recorded. (**d**) Baseline model, (**e**) low Na-channel density model. Failed dendritic spikes appear as small “spikelets” at the soma. (**f**) Gradient model. This model required 17% fewer Na-channels than the uniform density model to produce essentially the same propagation efficiency, but at the cost of lower excitability (fewer spikes). The modest steady increase in somatic spike height was due to increasing intracellular [Ca] which caused progressively deeper AHPs, leading to more Na-channel availability and higher spike amplitudes.

In other systems, impedance mismatches due to morphology can cause spike propagation to fail when dendritic Na-channel density is low [24]–[26]. Live recordings have shown that most ganglion cells initiate spikes in the axon/soma and actively propagate spikes into the dendrites, which do not initiate spikes [27], [28]. Thus the dendritic Na-channel densities of most retinal ganglion cells must be high enough to actively propagate spikes but not high enough to initiate them [28]–[30]. However, the DSGC initiates dendritic spikes, so starting from a Na-channel density considered normal for most ganglion cells, 25 ms/cm^2^, we set the Na-channel density high enough so that each dendritic spike successfully propagated to the soma and initiated a somatic spike (see Methods - Calibration, Figure 4a; [3]). To explore the requirements for successful dendritic spike propagation, we examined models with dendritic Na-channel densities lower than our calibrated models. In these low-dendritic-Na-channel models, synaptically-evoked spike propagation efficiency was low (Figure 4b, see Methods), because most spikes failed at a thick proximal dendrite branch-point, where they were attenuated by shunting from the large capacitance and low axial resistance. A linear density gradient with a higher proximal density of Na^+^ channels improved propagation (Figure 4c). Another consequence of this gradient was a smaller difference in spike threshold between proximal and distal dendritic regions (not illustrated). The F/I curve (see Methods) for somatic current injection was primarily affected by proximal dendritic Na^+^ density, and had a slightly lower slope for the gradient model, but still fit within the observed variability of physiological data.

We considered the conditions under which a sub-threshold depolarization could facilitate spike initiation. In the real DSGC, light stimulation by moving bars often produced a 5–10mV somatic depolarization 50–100ms prior to spiking. We found that propagation in models with low Na- channel density was also facilitated when somatic and proximal regions were depolarized either by injecting current at the soma or stimulating proximal regions with synaptic input. Transiently depolarizing proximal areas compensated for loss of current due to proximal high membrane conductance by bringing Na-channels closer to activation threshold. This suggested that a combination of proximal depolarization and high proximal Na-channel density could promote the successful propagation of dendritic spikes in the real DSGC.

### Dendritic regions differ in their spiking properties

**Figure 5 pcbi-1000899-g005:**
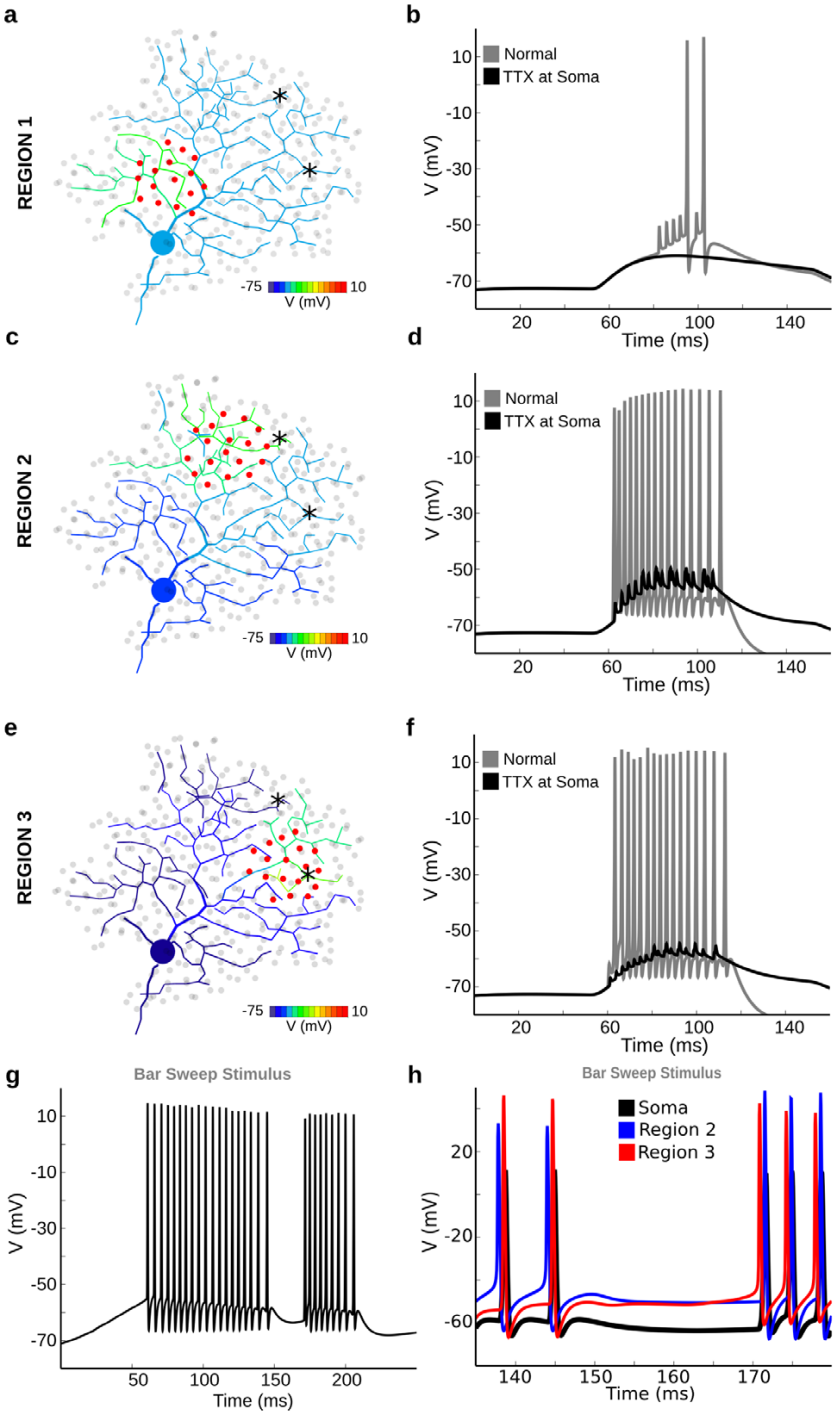
Dendritic regions differ in their spiking properties. A spot of light (60 µm dia) excited a small subregion of the On layer of the DSGC, under control conditions and during simulated focal application of TTX to the soma, which blocked Na-channels in the soma, thin segment, and proximal dendrites. Three local regions were examined in this figure. (**a**) Region 1 was selected for having high conductance thresholds. Excitation with a small spot produced local dendritic spike propagation, but an impedance mismatch caused spike failure upon reaching the primary dendrite. Recording that generated the color-map was taken at 80 ms, just before the first spikelet, to show the effect of the synaptic excitation. (**b**) Recording at soma, showing that excitation of region 1 in the model under control conditions (gray trace) produced dendritric spikes attenuated by propagation to the soma, seen as 10mV “spikelets”. When the spikelets sufficiently depolarized the soma, it initiated a full-blown spike. With somatic TTX application (black trace), no dendritic spikes were initiated because depolarization was insufficient. The reason was that somatic TTX application hyperpolarized the soma and proximal dendrites. (**c**) Region 2 was selected for having low-conductance thresholds and high propagation efficiencies. The recording was taken at 55 ms. (**d**) Excitation of region 2 produced large PSPs, dendritic spike initiation, and successful propagation to the soma, which initiated full-blown spiking. (gray trace). During somatic TTX application (black trace), dendritic spikes were initiated and appeared at the soma as small spikelets, attenuated in thick proximal dendrites that lacked active Na-channels. (**e**) Region 3 was selected for having slightly higher conductance thresholds than region 2, which therefore were less excitable. The recording was taken at 55 ms. (**f**) When stimulated, region 3 produced the same number of spikes as region 2, however the spike trains differed slightly in impulse shape and amplitude, and ISIs (gray trace). Simulated somatic TTX application (black trace) showed that dendritic spikes from region 3 were attenuated more than from region 2. The reason was that region 3 is farther from and therefore more isolated from the soma. (**g,h**) In a separate simulation, moving a bar from left-to-right across the dendritic arbor elicited spikes. Dendrites in region 2 were the first to spike, however when the leading edge of the bar crossed into region 3, the location of dendritic spike initiation moved to region 3. (**g**) Voltage recorded at the soma for the bar stimulus. The lack of spiking between ∼150ms and ∼170ms occurred when the leading edge of the bar was between region 2 and region 3, where not enough dendrites were depolarized sufficiently in either region to initiate a dendritic spike. (**h**) Voltage traces from the soma (black) and two locations in the dendritic tree, marked with asterisks in (**a,c,e**). The blue trace is from region 2, the red trace is from region 3. The relative timing of the spikes clearly shows that the region of dendritic spike initiation moved from region 2 to region 3 as the bar moved across the dendritic field. For each region, the smaller spikes were initiated first, and propagated to the other region as larger spikes which had a much faster rise from Vrest. The somatic spikes (black) arrived later because the soma was further along the path from the initiation site. The supplementary material contains a movie that shows spike initiation at a distal site, propagation to the soma, followed by backpropagation to the remainder of the dendritic tree (Video S1). Note that the cell is “winner-take-all”, i.e. whichever region has the strongest response will prevent other regions from spiking because each spike propagates to the entire cell, resetting its membrane voltage.

A previous study [3] indicated that dendritic spiking is responsible for the small spikelets seen in somatic recordings, but did not determine whether the spikelets represented full-blown dendritic spikes, or what parameters affected the distribution of spikelet amplitude. To explore these issues we ran a series of simulations in which a subregion of the dendritic tree was stimulated with a spot of light, and recordings made under normal conditions or with the Na-channels in the soma removed, thus simulating TTX application to the soma, which blocked somatic spiking as in the previous study (Figure 5). We found that each region had a characteristic excitability and ability to transmit spikelets of a certain amplitude to the soma, and that these properties varied across the dendritic tree (Figure 5a–f). Some regions were more sensitive than others and would spike more readily with a weak stimulus, and some regions were relatively insensitive to spiking. To test the effect of these differing excitabilities on a typical response, we ran a simulation of a bar passing over the DSGC, and recorded the somatic spiking and dendritic spiking in 2 locations (Figure 5g,h). The somatic spike train showed vigorous spiking separated by ∼20 ms where the bar passed between 2 regions of high excitability separated by a non-spiking region. As the stimulus passed across each region of high excitability, it initiated full-blown dendritic spikes that propagated to the soma and back-propagated throughout the dendritic tree (see Video S1). The previous study had shown that somatic PSPs during null direction stimulation, which were devoid of superimposed spikes, were often as large or larger than PSPs during preferred direction stimulation that produced vigorous spiking (see also Figure 1). We hypothesized that this directional difference was due to local inhibition that suppressed dendritic spike initiation in the null direction without reducing somatic PSP amplitude.

### Overlapping inhibition prevents spike initiation but not spike propagation

**Figure 6 pcbi-1000899-g006:**
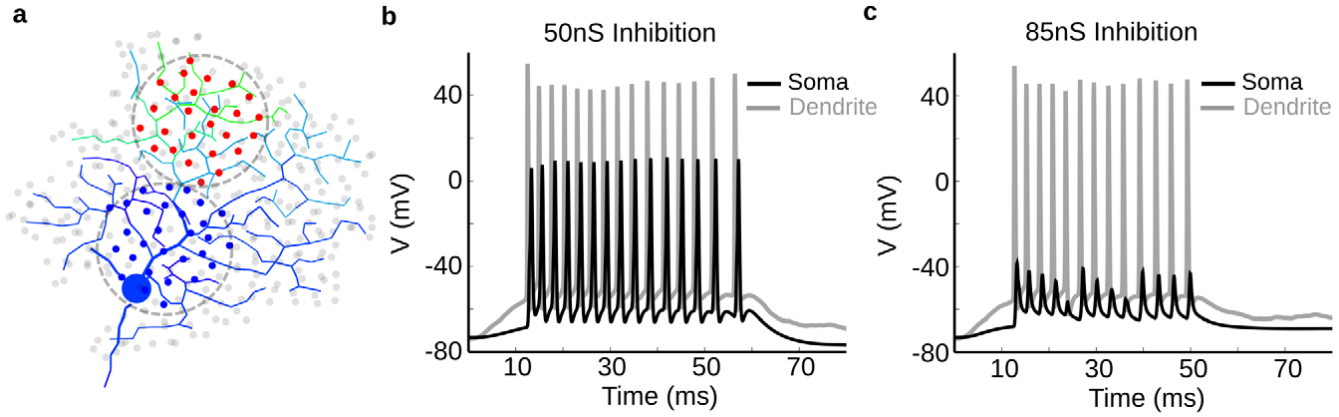
Unrealistically-strong on-path inhibition does not prevent dendritic spike propagation. (**a**) The model was stimulated by a spot of ∼30 identical excitatory inputs located in the distal dendrites (red dots), producing a peak conductance of ∼3nS. The strength of ∼30 identical inhibitory inputs (Vrev = −68 mV) located within a spot of the same size and placed close to the soma (blue dots) was modulated to explore the requirements for on-path shunting inhibition of dendritic spikes. Image was taken ∼10 ms after stimulus onset with the same color-map as Figure 2. None of the inhibitory synapses were located on the soma, and many activated by the spot (blue dots) were directly on the path for spikes propagating to the soma. (**b**) Recording from soma (black) and dendrite (gray) near the locus for spike initiation. A simultaneous inhibitory input with a peak total conductance of ∼50nS distributed equally amongst the blue synapses did not prevent dendritic spike propagation. This conductance is 2–5-fold larger than predicted from previous physiological measurements (see text). (**c**) When the total peak inhibition was raised even further (∼85nS), dendritic spikes (gray trace) were severely attenuated and appeared as small “spikelets” at the soma (black trace). Such events are rarely observed except during somatic TTX application [3].

We next tested the question whether inhibition functions in the DSGC dendritic tree mainly to prevent propagation of spikes, or whether it serves to prevent spike initiation. To explore the ability of inhibition to modulate the spiking properties of the DSGC, we ran simulations with different spatial arrangements of inhibitory synapses. Initially we wanted to evaluate how much “on-the-path” inhibition was required to suppress dendritic spike propagation. For these simulations we applied a 75µm “spot” of shunting inhibition (∼30 synapses, ∼300–3000 pS/synapse, reversal potential ∼V_rest_) over an area that covered the soma and proximal dendrites, while stimulating a distal region with a spot of excitatory input (∼30 synapses, ∼100 pS/synapse, reversal potential = 0mV,) (Figure 6a). Previous work has shown that the total peak inhibitory input to DSGCs is around ∼10nS [7], [31], however, given the limited visibility of synaptic currents for a somatic recording electrode, the actual inhibitory input to the dendrites will be somewhat larger (see Methods; [32]). We performed simulations in which we adjusted the magnitude of inhibition and excitation in the dendrites so that the conductance measured at the soma matched that recorded previously [7]. These results indicated that the actual synaptic conductance was likely to be about a factor of two larger than recorded at the soma (see Methods). Nonetheless we found that applying on-path inhibition of up to 5 times the observed values (50nS), even within a relatively small dendritic region, as suggested by prior theoretical work on non-spiking input [15], was insufficient to prevent dendritic spike propagation and produced only a modest attenuation in the spike amplitude (Figure 6b, black trace). Increasing the inhibition to 85nS did attenuate the dendritic spikes and prevent activation of a somatic spike. In this case, the dendritic spikes appeared at the soma as low amplitude “spikelets” (Figure 6c, black trace). We performed these simulations for excitation and on-path inhibition in several regions in the dendritic tree on several different cell morphologies, and all gave similar results showing that to be effective, the inhibition would have to be unrealistically strong. The reason, we found, was that to attenuate an actively propagating spike, the inhibitory conductance locally within the region of propagation must be larger than the peak activated Na-channel conductance. Further, we found that the precise timing of the spikes relative to inhibitory input over 50 ms was not important for blocking propagation, as long as there was substantial overlap [15], because the key factor was amplitude of the inhibitory conductance relative to the Na-channel conductance. The on-path inhibition also attenuated the dendritic PSP produced by excitatory input (Figure 6b,c gray traces). Given that dendritic spikelets are rarely observed at the soma of the DSGC during light stimulation [3], and that an unrealistically-high inhibitory conductance was needed to shunt propagating dendritic spikes, our conclusion from this set of experiments was that in the real cell, null-direction inhibition is much more likely to block spike initiation rather than spike propagation.

**Figure 7:**
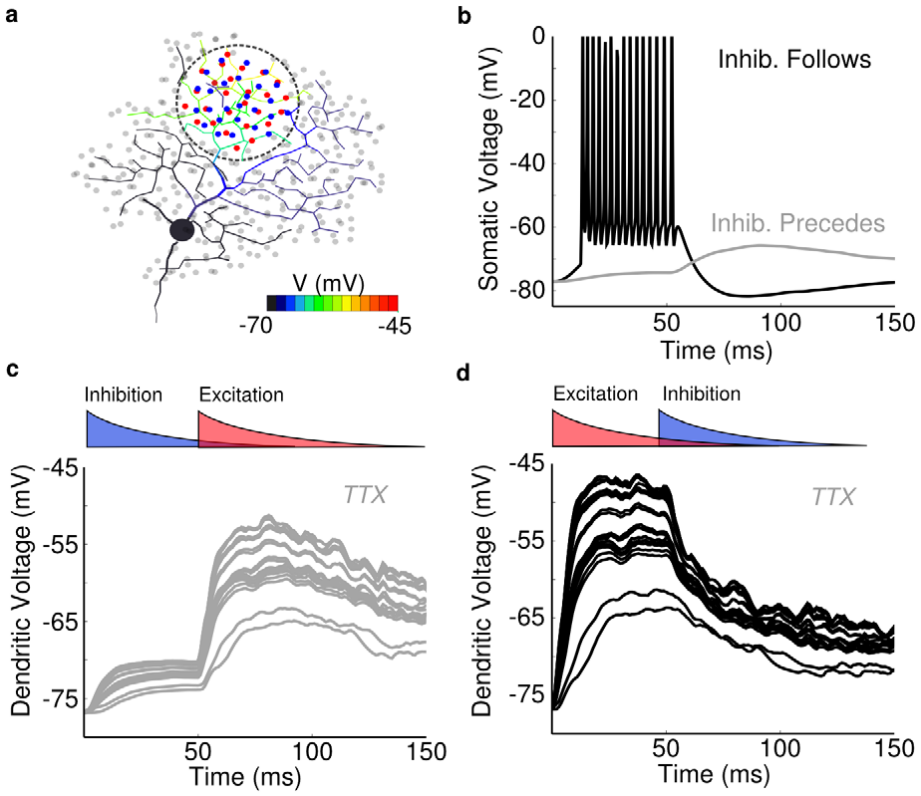
Prior inhibition can prevent spiking in co-extensive regions. Model with spot stimulus and temporally-offset inhibition. (**a**) Spot of activated synaptic input 75um in diameter at the On layer of DS060825 (red dots = excitation, blue dots = inhibition, time = ∼60ms). The spread of depolarization in the dendrites is shown in color, 5 ms after onset of excitation and 45 ms before inhibition, stimulus timing shown in inset of (d). Total peak excitatory conductance ranged from ∼1–4nS, inhibitory conductance ranged from 4–10nS. (**b**) Voltage recording from soma when inhibition followed (black) or preceded (gray) excitation. Preceding inhibition prevented the cell from spiking. (**c**) In a model lacking Na-channels, plots show voltage at several dendritic loci with inhibition 50ms before, or (**d**) after excitation. A comparison of the plots shows that delayed inhibition (d) allowed the dendritic PSPs to depolarize more than 5mV compared with leading inhibition (c). In the model with sodium channels (b), this difference was dramatically amplified by a nonlinear spike threshold. (c,d) Synapses were transient (decay time constant = 50ms). Overlapping filled red (excitatory) and blue (inhibitory) curves above PSPs show the time courses. The same simulation with a temporal offset of 25ms produced similar results. The small, initial rise in membrane potential following the onset of inhibition in (b) and (d) is due to the inhibitory reversal potential (−68 mV) being slightly more depolarized than the resting membrane potential. Each dendritic locus showed a different voltage response from its local excitation, the shunting inhibition, and the local input resistance.

We next wanted to determine how much inhibition was required to suppress dendritic spike initiation under the same conditions. The receptive field of the DSGC has both spatial and temporal components [2], [8], which are widely believed to result from spatially offset inhibition that trails excitation in the preferred direction. Because the DSGC's distal dendrites are electrotonically isolated, we hypothesized that a response observed in a local region could not represent electrotonic spread from synaptic inputs outside that region. Therefore, responses evoked in a local dendritic region would reflect the spatial localization of the stimulus, and not a time-delayed signal spreading from adjacent regions. To separate spatial from temporal effects within the local dendritic region, we first isolated temporal effects with a stationary, spatially distributed excitatory “spot” of synaptic input (dia = 50µm), preceded or followed by (Δt = −50 to +50 ms) a superimposed spot of inhibitory input (Figure 7a). Kinetic and conductance parameters of the synapses were selected so that excitatory and inhibitory conductances matched physiologically-observed values [7]. The excitatory synapses (200pS/synapse) and inhibitory synapses (275pS/synapse) both incorporated a transient temporal filter (τ = 50ms, high pass). In order to assess the required level of inhibition, we adjusted the amount of leading inhibition (i.e. arriving prior to excitation) in time (Δt = −50ms) to just prevent dendritic spike initiation (Figure 7b,d). When the temporal order was reversed, and inhibition was delayed, excitation depolarized the dendrites enough to generate spikes prior to the inhibition's onset (Figure 7c). Thus physiologically realistic levels of inhibition (4–10nS) can interact locally with excitation to produce a local directional difference in the PSP amplitude. A nonlinear spike threshold dramatically amplified this difference to produce a strongly direction-selective spike output (Figure 7a,b). We called this type of temporal processing the “postsynaptic DS” mechanism because it relied exclusively on interactions within the dendritic tree to generate DS. We next wanted to determine how well the model performed for a spatio-temporal stimulus, essentially identical to one that is regularly used for studying these cells.

### Directional differences are greater at dendritic tips

**Figure 8:**
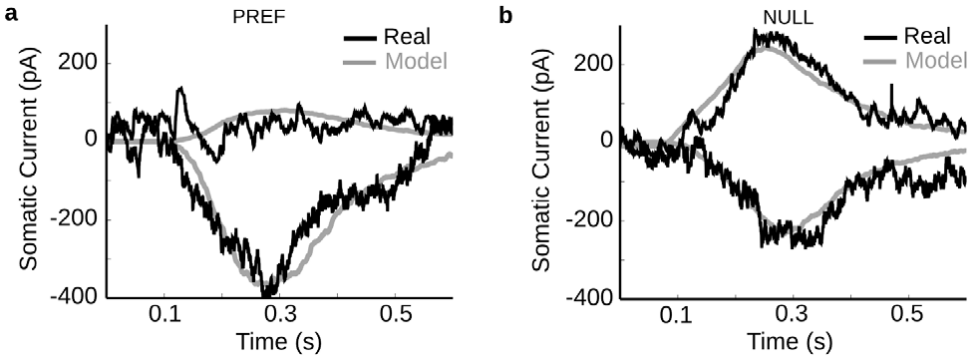
Calibration of model with direction-selective currents from a real DSGC. We simulated a bar stimulus moving in the preferred and null direction across the dendritic field, and adjusted the model parameters to reproduce the voltage clamp currents for the On-responses (cell DS060825). The shape of the responses derived from the dendritic morphology. (**a**) Real synaptic currents (black) measured by voltage clamp at the soma during preferred direction stimulation and recorded at holding potentials of −75mV (lower traces) and 0 mV (upper traces). The gray lines show the model predictions using a total excitatory conductance of ∼6.5nS and an inhibitory conductance of ∼2.5nS. Individual model inhibitory and excitatory synaptic inputs had the same time course (see Methods). (**b**) Same as (a) but for null direction stimuli. The outward current peaks ∼50 ms ahead of the inward current. This was accounted for by including a spatial offset of 50µm between inhibition and excitation, thus delaying inhibition in the pref direction (see text). The larger outward current and smaller inward current was accounted for by increasing the peak total inhibitory conductance to ∼6nS and reducing the peak total excitatory conductance to ∼3.5nS.

Previous work has shown that the excitatory and inhibitory inputs to DSGCs are already directional [6], [7], [16], [17], with inhibition being larger in the null than the preferred direction, and excitation being larger in the preferred than null direction. We explored how the model could reproduce the responses of the DSGCs under conditions where synaptic inputs were activated throughout the dendritic arbor according to the motion of the stimulus. For the “presynaptic” DS mechanism, the excitatory and inhibitory conductances at a dendritic locus varied with direction but were activated at the same time, and for the “postsynaptic” DS mechanism, the conductances remained constant with direction but were activated with an asymmetrical spatio-temporal offset. For the postsynaptic mechanism, we set inhibition with a spatial offset, to generate a temporal offset that was dependent on bar direction (See Methods). Stimulation in the pref direction activated excitatory synapses in advance of inhibitory synapses. As bar direction approached null, inhibition was set to overlap more with excitation, and completely overlapped excitation temporally and spatially in the null direction. Our baseline spatial offset produced inhibition that trailed excitation by ∼50ms in the pref direction. We also tested temporal offsets of 75ms, 150ms, and 200ms. When calibrating the model, we adjusted the spatial offset of the inhibition, and the magnitudes of the inhibitory and excitatory conductances so that the waveshape of the somatic currents measured with voltage clamp matched those recorded from a typical cell (Figure 8a,b; see Methods). To fit the currents in the preferred direction the total excitatory conductance was 6.5nS and inhibitory conductance was ∼2.5nS, while in the null direction excitation was 3.5nS and inhibition was 6nS. These preferred/null ratios of excitation and inhibition are within the ranges reported previously for DSGCs [7].

**Figure 9:**
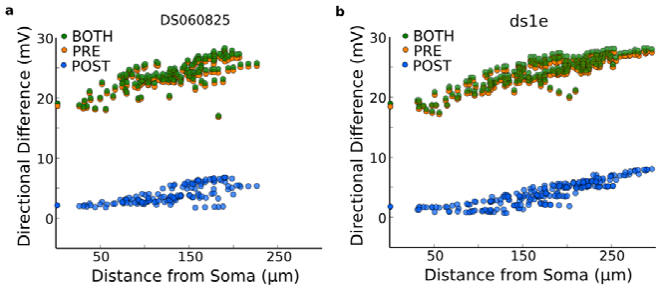
Presynaptic and postsynaptic DS mechanisms vary with dendritic locus but are more robust when combined. For 2 morphologies, (**a**) DS060825, (**b**) ds1e, Na channels were removed from the model to simulate bath TTX application, and a bar stimulus moved from left-to-right (Pref) and then right-to-left (Null). During bar movement dendritic PSPs were recorded from points across the dendritic tree. The directional difference (DD) for a given dendritic location was the difference in PSP amplitude between a Pref and Null bar stimulus. For both morphologies, the presynaptic mechanism (orange) produced a robust increase in dendritic PSP. The postsynaptic mechanism (blue) also successfully overrode the intrinsic DS of each distal dendrite. When the mechanisms were combined (green), they cooperated to produce greater DDs. Although this modulated the DD amplitude and gave a greater temporal difference between excitation and inhibition, it did not increase the overall DD much because the presynaptic mechanism was stronger. Distal regions had a higher directional difference than proximal, and the greatest directional differences occurred for rightmost dendritic tips (on the Null side) where the intrinsic DS matched the preferred direction.

Once calibrated, we measured the directional-difference in the PSP amplitude for a model without Na-channels as a function of the distance from the soma (Figure 9). The simulations included either the presynaptic mechanism from Figure 8, where both amplitude and waveshape of the PSPs depended on direction, the postsynaptic mechanism, where only the temporal offset between excitation and inhibition depended on direction and the amplitudes did not vary, or both mechanisms. The results showed that the directional-difference was largest in the peripheral dendrites, which also corresponded to the areas of highest excitability (Figure 3). The model reproduced the relatively small directional-difference in the somatic PSP amplitude that is seen in real recordings (compare Figure 3d and Figure 1). The magnitude of the postsynaptic mechanism was largest for the peripheral dendrites but dropped to almost zero near the soma (Figure 9). The reason was that the magnitude of the postsynaptic mechanism was directly related to the input resistance and the PSP amplitude (Figure 2).

### Dendritic structure produces an intrinsic DS signal that is strongest in distal regions

**Figure 10:**
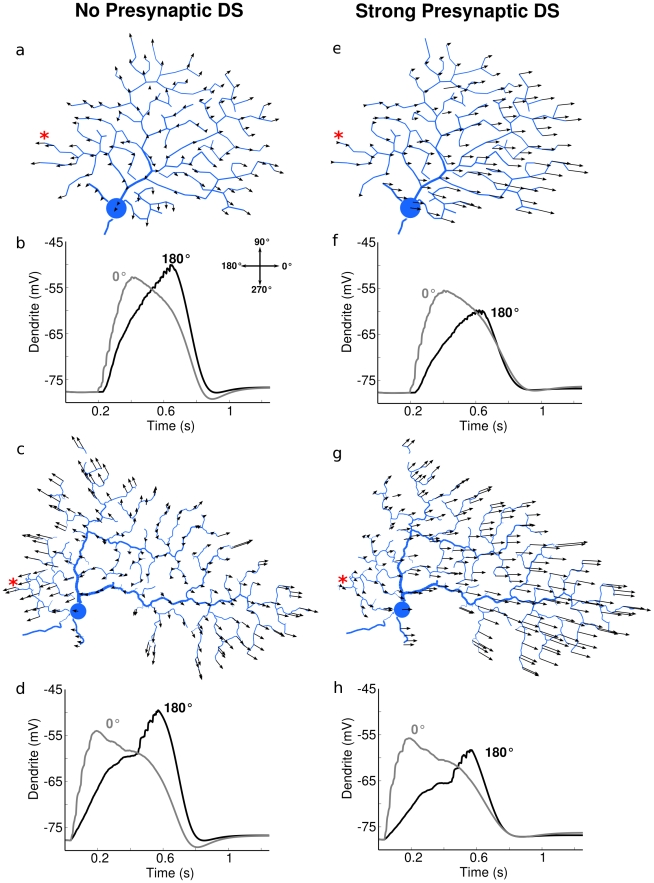
The presynaptic DS mechanism overrides intrinsic DS in preferred-side distal dendrites. In models with Na-channels blocked, PSPs were recorded (black arrows) in response to a bar swept in 8 directions (0°–315°, increments of 45°) across the dendritic field. Two synaptic input configurations are shown; one with equal excitation in each direction and no inhibition (left column, “No presynaptic DS”), and another where excitation was maximal at 0° and minimal at 180°, and inhibition varied in the opposite manner (right column, “Strong presynaptic DS”). (**a**) The DS index and angle for the PSP at each dendritic point were computed for bar sweeps with symmetric synaptic input (see Methods). This unmasked an “intrinsic DS” for distal dendrites that pointed radially outward, with DS indices ranging from 0.01 to 0.07. (**b**) The voltage recorded from the dendritic point (on the preferred side) marked with a red asterisk. The PSP evoked by movement in the direction of the intrinsic DS was higher than in the opposite direction. (**e,f**) DS index and angle for each dendritic point were measured in the same way, except with the addition of presynaptic DS. (**e**) The presynaptic input overrode the intrinsic DS for all points. (**f**) The voltage at the same point as in (b), showing that presynaptic DS effectively masks the “intrinsic DS” of the dendritic point. (**c,d,g,h**) The same simulations performed for another DS morphology.

To determine the contribution of the DSGC's morphology to its direction-selective response, we ran simulations with a moving bar in a simplified model without Na-channels that included only excitatory synaptic input to the DSGC that did not vary according to bar direction, while recording responses at the soma and throughout the dendritic tree. We measured the DS index (0 = non-directional, 1 = fully directional; see Methods) and vector angle of the dendritic PSPs evoked by a bar stimulus that moved alternately in eight directions, and found that the distal dendrites had a weak “intrinsic DS”, with preferred directions that pointed radially outward from the approximate geometric center of the dendritic arbor (Figure 10a,c). This intrinsic DS resulted from spatial summation in dendrites similar to that described in models of starburst amacrine cells [33]–[36]. The directional asymmetry results from partial isolation between a dendritic compartment and the soma, which delays summation of the somatic PSP with the dendritic PSP during centripetal motion [34]. Because the somatic response represents the summation of PSPs from all the dendrites, the effects of the intrinsic DS tend to cancel out resulting in little intrinsic DS measured at the soma. However, the responses in most of the distal dendrites were clearly direction-selective, tuned to the centrifugal direction.

### Interaction between presynaptic and intrinsic DS mechanisms

To explore the interaction between presynaptic DS and intrinsic DS, we configured the bar stimulus with the above-described “presynaptic DS” mechanism, where excitation was strong in one direction (0°) and weaker in the opposite direction (180°). Inhibition for this input was set to be the opposite, weakest when the bar moved at 0° and strongest at 180°. We then ran a series of simulations as before, one for each dendritic location, measuring the DS index and vector angle. On the null side of the dendritic tree (closest to an advancing null stimulus) where the intrinsic DS of the distal dendrite agreed with the presynaptic DS, the directional difference of the PSPs was 2-fold or more that observed without presynaptic DS (Figure 10a,c,c,g), On the pref side of the dendritic tree (the side from which a preferred stimulus originates), the results showed that the presynaptic mechanism can override the intrinsic DS, producing a directional difference in the evoked PSPs opposite to the local intrinsic DS signal (Figure 10b,f,d,h).

This analysis demonstrates that the intrinsic DS at each dendritic location can be large enough to enhance or reduce the local directional difference in the PSP amplitude produced by addition of the postsynaptic and presynaptic DS mechanisms (Figures 9, 10). The intrinsic DS mechanism enhanced the DS responses on the null side of the dendritic arbor, and conversely, weakened DS signals on the preferred side of the arbor (Figure 10). It is interesting to note that there is a well documented “non-DS” zone located on the preferred side of the DSGC [2], [37], within which directional responses are much weaker or even absent. These results suggested that the effects of intrinsic dendritic DS may account for the non-DS zone.

### Dendritic spikes amplify the DS index of PSPs

**Figure 11:**
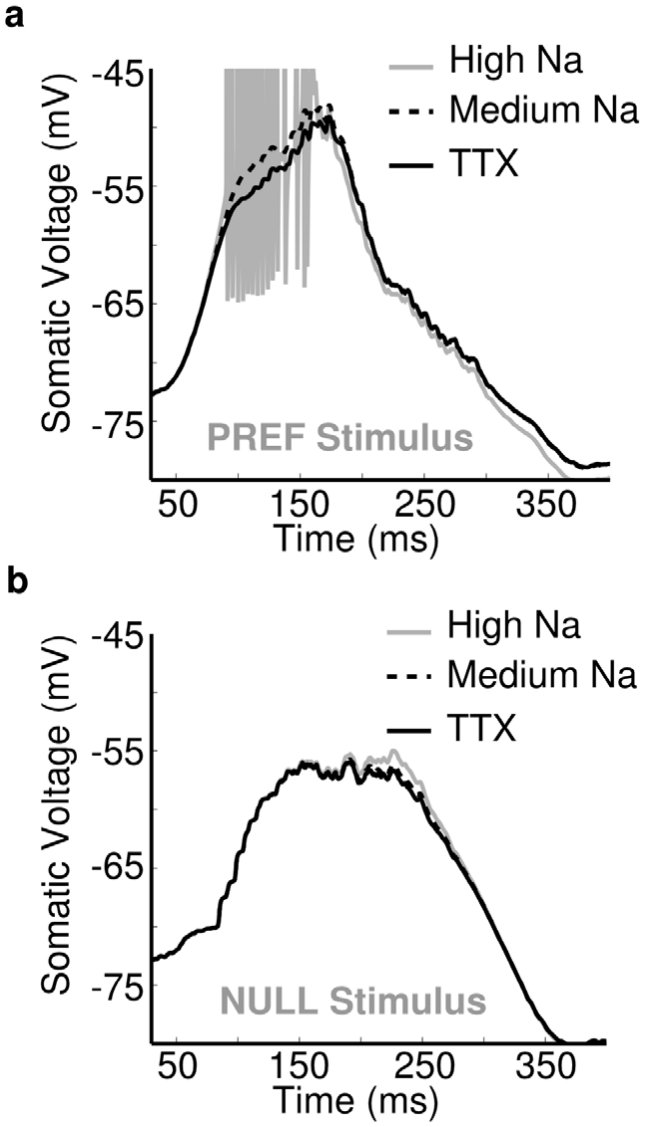
Na-channels amplify the directional difference in PSPs. Dendritic PSPs were examined in models with a high dendritic Na-channel density (gray, g_Na1.6_ = 40mS/cm^2^), low density (dashed, g_Na1.6_ = 10mS/cm^2^) and no Na-channels (black, TTX). (**a**) PSPs recorded at a typical point in the distal dendritic tree. The Na-channels at in the medium density model amplified the PSPs in subthreshold mode, producing a larger PSP than for the TTX model (∼1–2mV). With a higher Na-channel density the stimulus elicited dendritic spiking. KCa channel activation from spiking resulted in a lower voltage on the falling edge of the response. (**b**) Traces from the same three models for a bar during null-direction stimulation showing weaker amplification. Maximum difference in peak PSP between TTX and medium, 0.5mV, and between medium and high, 1.2mV. Peak total excitatory conductance was ∼3nS, peak total inhibitory conductance was ∼5nS.

**Figure 12:**
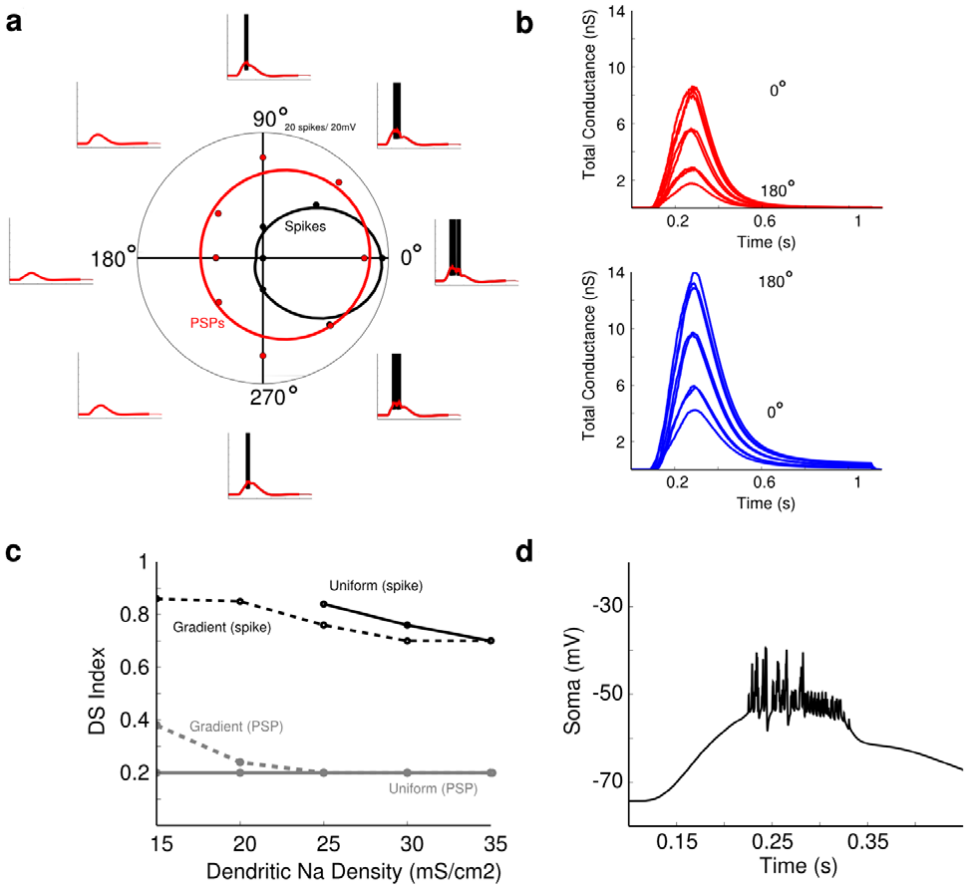
Dendritic spikes amplify DSI recorded from spikes and PSPs at the soma. Responses to moving bars were simulated as for Figure 10 with the same preferred direction (0 deg). Excitatory and inhibitory synapses were distributed throughout the dendritic arbor. Presynaptic DS excitation was generated by setting the peak conductances (excitation 9nS pref, 2nS null; inhibition 4nS pref, 14nS null). We then measured the amplitude of the response and the DS index for the spikes and the underlying PSPs (see Methods; [3]). (**a**) Polar plots of the number of spikes (black) and peak PSP amplitude (red) as a function of stimulus direction, fit to von Mises functions (circular Gaussians). The surrounding traces show the spikes (black), and the PSPs (red). (**b**) The time courses for the simulated excitatory (red) and inhibitory (blue) synaptic conductances (see Methods). (**c**) The DS indices calculated for spikes (black), and PSPs (gray) were measured in models having different dendritic Na-channel densities. One type of simulation included a spatially uniform dendritic Na density (solid lines) in the range of 10–35mS/cm^2^, and another included a spatial gradient (dashed lines), where the proximal density was fixed at 35mS/cm^2^, while the distal density ranged from 10–35mS/cm^2^. The DS index for spikes was higher than for PSPs in simulations with the Na-channel gradient, and for most with a uniform density. As dendritic Na-channel density was reduced, less spikes occurred in the null direction, which increased the DS index. The gradient model provided a high DS with a slightly lower number of dendritic Na-channels. (**d**) Simulated TTX application to the soma that blocked Na-channels at the soma and proximal dendrites. The response included PSPs with superimposed dendritic spikelets which propagated from the distal dendrites to the soma. The measured DS index was 0.50, significantly higher than the DS index for PSPs at any dendritic Na density.

When Na-channels were included, the model reproduced the DS spiking response of the cell. The Na-channels amplified the preferred PSPs more than null PSPs within a local region because the preferred PSPs were more depolarized (Figure 11). We tested the effect of different Na-channel densities, and found that this selective amplification effect occurred in both sub-threshold mode and when spikes were initiated (Figure 11). The spike threshold within local dendritic regions effectively amplified the directional difference of the PSPs to produce strongly direction-selective somatic spikes. To determine the role of dendritic spiking relative to the other DS mechanisms identified above (presynaptic, postsynaptic, and intrinsic), we simulated a bar passing over the DSGC in different directions, and measured the magnitude of the spike and PSP responses and their DS index (Figure 12a). We adjusted the excitatory and inhibitory inputs so that the DS index of the PSPs was ∼0.2 (Figure 12b, similar to that recorded from real cells), and found that the DS index of the resulting spikes was ∼0.8, about 4-fold higher than for the PSPs (Figure 12c). We measured the DS index with different Na-channel densities and in addition compared them to a uniform density with a gradient. Higher Na-channel densities, although they tended to generate more spikes, did not increase the DS index. Instead, the lower densities and the gradient gave a higher DS index, because they gave a greater difference in spiking between preferred and null directions. We simulated local TTX application to the soma, as was done experimentally [3], by turning off somatic Na-channels. The DS index of the resulting spikelets was 0.5, which was higher than PSPs alone but lower than for full-blown somatic spikes (Figure 12d). This implied that, besides carrying dendritic signals to the soma, the role of spikes is to amplify the directional difference of the PSPs received by the DSGC, and that direction-selective spiking is generated at least in part by postsynaptic non-linearities. We next considered the interactions between the presynaptic and postsynaptic mechanisms.

### Presynaptic and postsynaptic mechanisms cooperate to increase overall DS index

**Figure 13:**
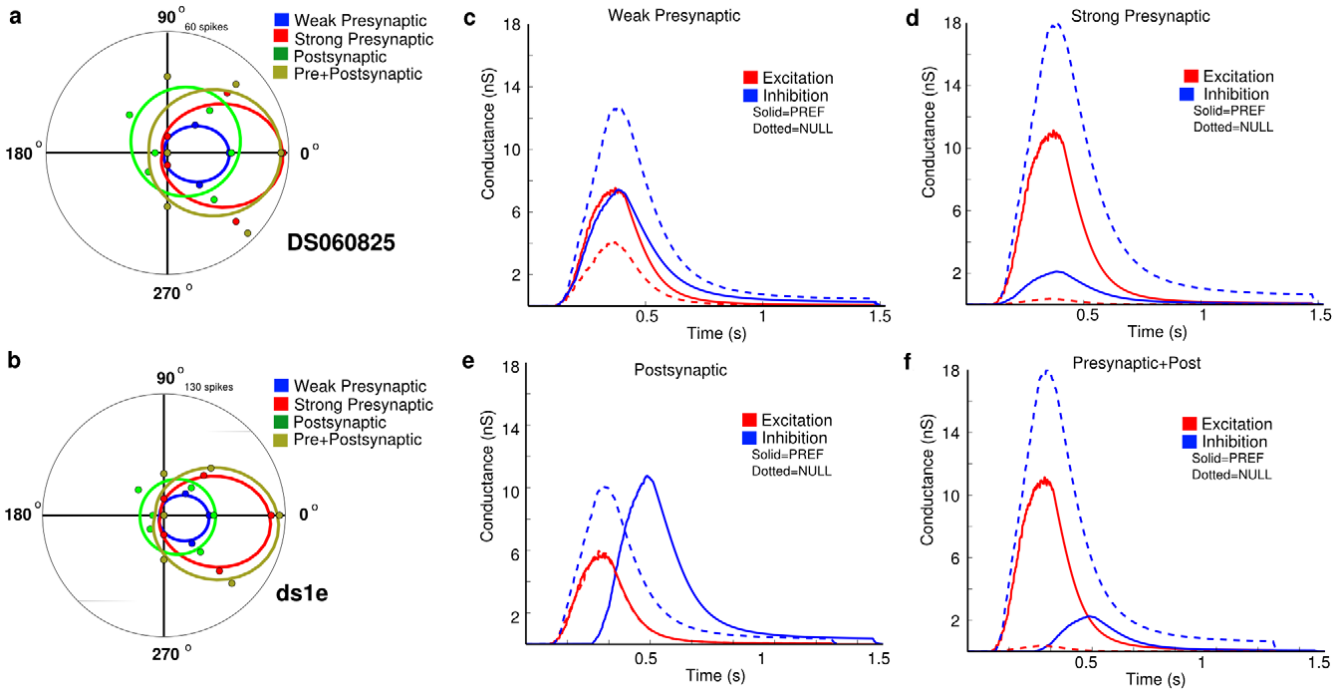
Presynaptic and postsynaptic mechanisms cooperate to increase overall DS index. (**a,b**) Directionality of the spike response for two morphologies, DS060825 (a) and ds1e (b), which were given the same synaptic input configurations, fitted to von Mises functions. For each simulation, 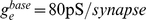 and 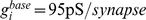. We recorded the spike response in each direction for four different synaptic configurations. (**c,d**) For the strong presynaptic (a,b, red) 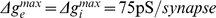, for the weak presynaptic (a,b, blue) 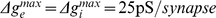. (**e**) For the postsynaptic mechanism (a,b, green) inhibition was coincident with excitation in the Null direction, and trailed excitation by ∼150µm in the Pref direction, while the strength of excitation and inhibition were held constant in each direction. (**f**) In the presynaptic+post configuration (a,b, orange), the strong presynaptic mechanism was combined with the postsynaptic mechanism (Figure 4 in [7]). Overall, the presynaptic mechanism was more effective than the postsynaptic mechanism for preventing spikes in the Null direction, but the postsynaptic mechanism produced a higher overall number of spikes. The combined mechanisms produced less spikes in the Null direction and more in the Pref, and a higher overall DS index. The ds1e morphology (b) also produced a higher overall number of spikes due to a higher dendritic density.

We simulated the presynaptic and postsynaptic mechanisms independently and then combined them to explore how each one contributes to produce directional-differences in dendritic PSPs and to direction-selective spiking (Figure 13). The strength of the synapses was set to produce peak excitatory and inhibitory conductances within physiologically-observed ranges [7]. As above, we simulated the presynaptic DS mechanism by modulating the time-course of the synaptic conductances, and the postsynaptic mechanism with spatially offset inhibition. For simulations with active Na-channels and either presynaptic or postsynaptic mechanisms alone, spiking was strong in the preferred and weak in the null direction (Figure 13a,b), but the presynaptic mechanism produced a stronger DS index than the postsynaptic mechanism (Figure 13a,b). When both mechanisms were combined, the DSGC again spiked in the preferred direction but not the null, and the DS index was the greatest. Thus, pre- and postsynaptic mechanisms cooperated to produce directional differences in the dendrites (Figure 12), which were then non-linearly amplified with a spike threshold to produce the DSGC's spiking response (Figure 13).

### DS is robust with changes in space constant

**Figure 14:**
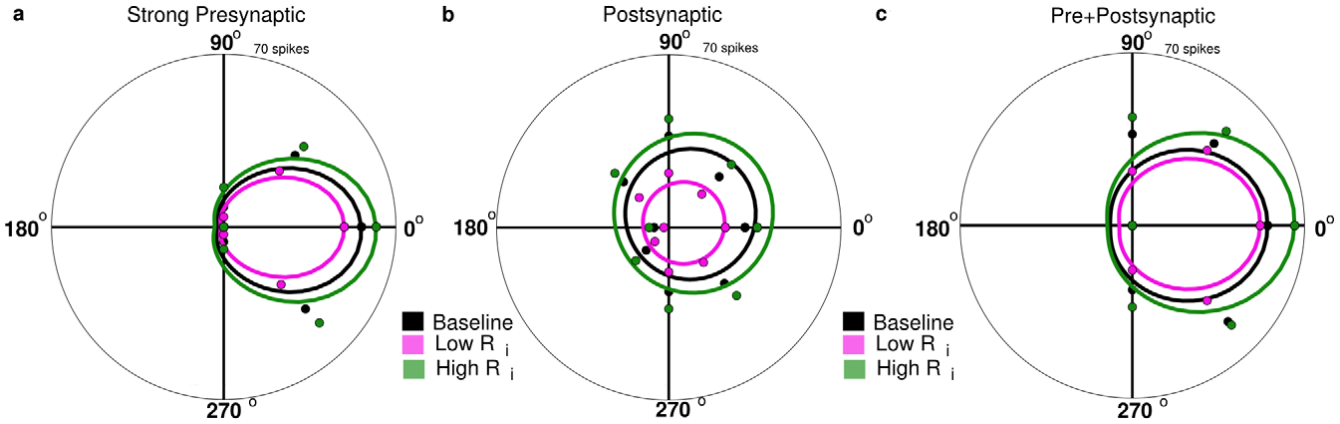
Direction selectivity is robust despite changes in space constant for both presynaptic and postsynaptic mechanisms. We varied the axial resistance (Baseline R_i_ = 200 Ω-cm, High R_i_ = 275 Ω -cm, Low R_i_ = 125 Ω-cm) and recorded spike responses for bar movement in 8 directions. Responses are shown here as polar plots for the strong presynaptic (a) and postsynaptic (b) mechanisms, for the DS060825 morphology. (**a**) A high axial resistance (green) caused less axial leak of current, and therefore greater amplification of the synaptic input and a higher overall spike rate, while low axial resistance (pink) had the opposite effect. The overall shape of the polar plot was not altered. (**b**) For the postsynaptic mechanism, a high axial resistance increased the overall number of spikes, as in the presynaptic mechanism, but also altered the shape of the polar plot, and produced relatively more spikes in the Null direction. (**c**) When both mechanisms were included, the result showed more spikes in the Pref direction and greater direction selectivity than the postsynaptic mechanism alone. A high axial resistance diminished the ability of leading inhibition to shunt sub-threshold voltage changes, and therefore more spikes were produced. Decreasing axial resistance had the effect of diminishing spikes in the Null direction and sharpening DS, because it enhanced the shunting ability of leading inhibition.

Finally, our morphological models inevitably contain uncertainties as to the dendritic diameter and the surface membrane resistivity that could affect the dendritic space constants, which in turn can influence the degree of dendritic isolation. Because the findings presented here predict that dendritic isolation within the DSGC is an important biophysical factor for generating its directional selectivity, we explored how the DS response was affected by changes in the space constant of the dendritic tree. We ran simulations in 8 different directions with different values of the dendritic axial resistance (Ri). A high value of axial resistance diminished the spread of axial current through the dendrites, which decreased the space constant (Figure 2) and amplified the presynaptic DS mechanism without changing the relative responses in different directions (Figure 14a). A high value of axial resistance also diminished the effect of shunting by the leading inhibition of the postsynaptic mechanism, increasing the number of spikes in both preferred and null directions (Figure 14b). A reduced value of Ri had opposite effects. When both mechanisms were combined, the resulting directional selectivity was intermediate between that for the presynaptic or postsynaptic mechanisms alone (Figure 14c).

### Somatic PSPs lack directional correlation with spikes

**Figure 15:**
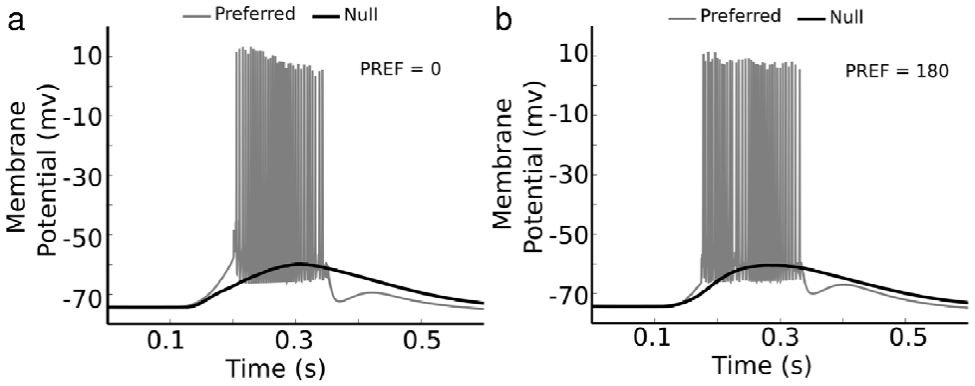
Somatic PSPs and spikes are uncorrelated because PSPs are attenuated by dendritic tree (compare with Figure 1a). (**a**) Simulation of a bar moving across the dendritic tree with a weak presynaptic+post synaptic mechanism, where the preferred direction was set to be 0 degrees (see Figure 14). Peak somatic depolarization is greater for Null (black) than Pref (gray). In the Pref direction, distal dendrites were depolarized enough to spike, and the dendritic spike propagated to the soma and initiated a somatic spike, despite the somatic PSP being below spike threshold. In the Null direction, distal dendritic PSPs were insufficient to elicit a dendritic spike, and the somatic PSP did not reach spike threshold, so no spikes were generated. (**b**) The same simulation, but with the preferred direction changed to 180 degrees, showing the effect of the asymmetrical dendritic tree on the PSP rise time, shape and spiking. The apparent difference in initial spike threshold between (a) and (b) reflects the underlying PSPs, i.e. in (a) the preferred direction the underlying PSP rose more quickly. The simulation was run on the same morphology as Figure 2c (DS060825), with bar velocity 1000 um/s, excitatory conductance 75pS/synapse, inhibitory conductance 95 pS/synapse, presynaptic mechanism (difference in conductances) reduced to 25% of the mechanism from Figure 8.

Once we had developed intuition about how the dendritic tree attenuates PSPs but not spikes, the apparent paradox of Figure 1 was straightforward to understand. A simulation of a somatic recording duplicated the lack of correlation between the PSP amplitude and spiking (Figure 15). From the previous simulations, we learned that spikes propagate from the dendrites and depolarize the somatic voltage quickly enough to initiate somatic spikes, even from a membrane potential hyperpolarized below spike threshold by 5–10 mV (Figures 4–7). The dendritic spike is not visible because the somatic spike overlays it precisely [3] (Figure 4). The recordings shown in Figures 1 and 15 show the result of somatic spiking summed with a compound PSP generated by synaptic conductances across the dendritic tree. From inspection of the spikes in the preferred direction (gray trace), the after-hyperpolarization (bottom envelope, Figure 1, 15a) of the spikes progressively depolarizes by a few mV through each spike burst. The explanation is that the origin of the PSPs and thus their relative amplitude changes depending on the position of the moving bar. The first spikes start when the bar passes over the distal tips of the dendrites. The corresponding somatic PSPs are attenuated by a few mV (Figure 2f). Later spikes in the burst initiate from more proximal dendritic regions, and the corresponding PSPs are less attenuated at the soma. Note, however, that this somatic recording does not reflect the amplitude of the distal PSPs – they are unattenuated by electrotonic decay and thus have a large directional difference to initiate robust spiking.

The recordings from the null direction of Figures 1 and 15 (black trace) show a compound PSP with greater amplitude but without initial spiking. These recordings reflect PSPs from a more proximal dendritic location that are less attenuated than from a more peripheral dendritic location. The PSPs from this more proximal region are insufficient to cause local spiking because they are shunted by the proximity to the soma. Although the null direction PSPs initiate hardly any spiking, they propagate without much attenuation to the soma and so appear larger than the preferred direction PSPs. Further, because the soma is hyperpolarized 5–10 mV below spike threshold, any dendritic PSP that propagates toward the soma also tends to be attenuated and hyperpolarized, reducing the probability that it will reach spike threshold after back- propagating distally.

## Discussion

Our results provide a strong rationale for the role of several mechanisms in processing of direction-selective signals by the direction-selective ganglion cell (DSGC). They imply that the electrotonic properties of DSGC dendrites partition the cell into separate computational regions, each of which sums its local excitatory and inhibitory synaptic inputs, and initiates spikes when the local spike threshold is exceeded (Figures 2–6). They further imply that the role of dendritic spiking in the DSGC is several-fold. First, the nonlinear spike threshold effectively amplifies the directional difference in the PSP response amplitude within local dendritic regions, thereby enhancing the directional tuning of the cell's response (Figure 13) [38]. Second, dendritic spikes are necessary to propagate the DS signal from the separate computational sub-regions to the soma and axon (Figure 2). Third, during a propagating dendritic spike, the aggregate Na-channel conductance along a dendrite is large enough to give each spike a high probability of reaching the soma and axon, thereby preserving the direction-selective signal in the presence of ongoing synaptic activity in other regions of the dendritic arbor (Figure 6).

In their original description of the DSGC, Barlow and Levick [2] noted that direction-selective spike output was produced for stimuli that activated only a small fraction (<20%) of the total synaptic input to the cell. They proposed that the synaptic mechanism comprised “subunits” that could compute DS locally, and were repeated numerous times across the dendritic arbor. Later workers showed that these subunits might be even smaller [39]. An obvious problem with the existence of such subunits is that stimulation of a small fraction of the total inputs will produce concomitantly small somatic PSPs, and thus it is difficult to envisage how a somatic spike threshold could produce directional selectivity across a broad range of stimulus configurations.

The modeling and theoretical analysis presented here provides an explanation for the “subunits”, by showing that DS subunits are an inevitable result of the electrotonic properties of the DSGC dendritic arbor. The predicted attenuation of PSPs between the dendrites and the soma renders direction-selective spike initiation at the soma untenable, and in real neurons this will be exacerbated for small movements over the distal dendrites. The data in Figure 1 neatly illustrates the phenomenon, and shows that somatic membrane potential does not drive the spiking output [3]. Local dendritic spike initiation overcomes this problem, and allows for greatly enhanced direction sensitivity. Although a stimulus with limited motion over a distal dendrite will produce a weak directional difference in somatic PSPs due to attenuation from dendrite to soma, it will produce strong DS in local dendritic spiking , in part due to the high local input resistance, and thus strong DS spiking at the soma. Moreover, sensitivity will be enhanced for full-field stimulation, because as an edge moves across the entire dendritic arbor, DS spikes will be initiated at numerous points within the dendrites. If DSGCs had thicker dendrites and the somatic PSP reflected a less attenuated summation of inputs across dendritic arbor, then it would inevitably lose sensitivity for small objects and small motions. An unexpected outcome of the model was the explanation for the presence of a non-DS zone within the dendritic arbor of the DSGCs [2], [37], a phenomenon that has not previously been adequately accounted for. Our results show that the non-DS zone is a consequence of local dendritic processing superimposed upon an inherent asymmetry that is predicted to be present within the dendritic arbor of every neuron [34].

### Rationale for presynaptic DS

Our simulations indicate that local dendritic processing follows from the dendritic structure, and that a purely postsynaptic model can produce strong directional signals (Figure 13). One might then ask why presynaptic mechanisms have also evolved. Without presynaptic computation of DS, the directional selectivity of the DSGC would suffer because the postsynaptic mechanism decays to almost zero near the soma (Figure 9) and is reduced on the preferred side of the dendritic tree by the intrinsic DS within the dendrites (Figure 10). Thus presynaptic mechanisms can overcome limitations inherent in postsynaptic processing and produce a more robust system. However, the presence of a non-DS zone in many cells suggests that in many cases presynaptic mechanisms are not strong enough to overcome the intrinsic dendritic bias. This is consistent with a previous report showing that the strength of the presynaptic DS signal is very variable across the population of cells [7]. Clearly a relatively strong presynaptic mechanism would produce a strong and consistent DS signal at the soma (Figure 9). Our results predict that the variability in the strength of the presynaptic DS signal will be correlated with the variability in the strength of DS in the somatic PSP, with cells having a relatively weak presynaptic DS component also displaying weak DS in somatic PSPs, as illustrated in Figures 1 and 15. Further work will be required to fully explore the interactions of presynaptic and postsynaptic mechanisms in the DSGC. The circuitry that generates the presynaptic DS is currently under intense scrutiny and is beyond the scope of this study.

### Dendritic winner-take-all

One of the consequences of dendritic initiation of spiking, revealed by the simulations, is that when a dendritic spike reaches the soma it will spread throughout the entire cell (see Video S1) and obliterate any other simultaneous dendritic spikes [40]. The result is that the dendritic region with the lowest spike threshold will dominate the responses of the cell, because that region will reach threshold first, and therefore will also recover from the ensuing refractory period first, giving a role of “winner-take-all” to the most excitable regions (Figure 3). The occurrence of dendritic “hot-spots” was predicted by models in which identical synaptic inputs are distributed across the dendritic arbor (Figure 3d–f). Such results raise the question whether the responses of DSGCs are dominated by inputs from only a few dendritic regions, or whether cellular mechanisms exist that even out sensitivity across the dendritic arbor so that dendritic spike initiation is equally likely from any point. Although the answer to this question is unknown, the results of live recordings suggest that typical DSGCs initiate spikes in only a few local regions [3]. Our tests of density gradients in Na^+^ channels suggest that the excitability could be regulated by a nonuniform density of Na- and K-channels (Figures 3,4).

### Error in somatic measurements of dendritic conductance

One criticism of voltage-clamp recordings of neurons having synaptic inputs on an extended dendritic tree, especially the DSGC in which dendritic tips are isolated from the soma, is that estimates of conductance are inaccurate because the cell is not adequately space-clamped. To determine how accurate measurements of conductance are in cells of this type, we simulated voltage-clamping the soma and measured synaptic conductances according to the established protocol [6], [7]. These simulations indicated that estimated conductances differed from the actual ones by 50–100% (see Methods). The accuracy of the estimate of excitatory conductance was greater than that of the inhibitory estimate because voltage clamp errors were greater at depolarized clamp potentials due to axial resistance and the relatively hyperpolarized dendritic membrane, leading to a reversal potential more positive than expected.

These simulation results emphasize that a major advantage of computational models is the ability to look closely at mechanisms that would be difficult to study in the real neural system. The model allows the experimenter to estimate a range of errors, taking into account the accuracy of the data provided, and to identify what mechanisms in the neural system are responsible for the errors. Thus, with the dendritic morphology and a few simple assumptions and measurements, the actual conductances can be determined with a greater accuracy.

### Realism of the model

Because our results depend on a theoretical model, it is reasonable to ask how relevant they are to the real neural circuit. The simulations were sequentially calibrated, starting first with spike shape and amplitude (see Methods), then excitability with injected current (F/I plot), and finally proceeding to higher level tests of the spikelet amplitude and behavior. Although the original morphology was derived from careful measurements, in most cases from confocal stacks, some imprecision in the diameters of the reconstructed dendrites is inevitable. We took this into account by bracketing the diameters using an additional multiplicative factor in the models, then verifying that the overall dendritic surface area and time constant were correct by matching the charging curve with injected current. We verified that the results did not depend on a unique combination of parameters, for example, the particular morphology of the dendritic tree, or some unique combination of channel types or their densities - all of our conclusions are based on phenomena that emerged from the simulations. For example, the intrinsic weak DS found in the dendritic system, although derived from the morphology and biophysical membrane parameters, was robust and did not depend strongly upon a particular choice of model parameters (see Methods).

### Importance of local dendritic processing for the brain

The local initiation of dendritic spikes described here that propagate with high probability to the soma represents a general mechanism for performing independent nonlinear computations leading to a decision [41]. For example, a complex cortical cell sums signals nonlinearly from its presynaptic neurons [42]. The synaptic signals originate from a large number of presynaptic neurons, and the amplification performed in any local subregion by nonlinear summation of PSPs in subthreshold mode can independently amplify the signal, potentially leading to a spike [39]. The spike generated by this process can override the processing of other local regions along the propagation route. When a dendritic spike propagates to the soma and axon it provides the neuron with an all-or-none decision based on the nonlinear processing performed by any of the independent local computational subunits [43].

## Methods

### Tissue preparation and maintenance

Experiments were conducted in accordance with protocols approved by the Institutional Animal Care and Use Committee at Oregon Health and Science University and NIH guidelines. Dark-adapted, pigmented rabbits were surgically anesthetized with sodium pentobarbital and the eyes removed under dim-red illumination. The animals were then killed by anesthetic overdose. All subsequent manipulations were performed under infrared illumination (>900nm) or under dim red light (>620nm). The anterior portion of the eye was removed and the eyecup was transected immediately above the visual streak. The ventral piece was used exclusively in all experiments. The retina was dissected from the eye, and a 5 by 5 mm section of central retina was adhered photoreceptor side down, to a circular glass cover-slip coated with poly-L-lysine (Sigma) or Cell-Tak (BD Bioscience, USA) and placed in the recording chamber (∼0.5 ml volume). The preparation was continuously perfused (∼4 ml/min) with oxygenated bicarbonate-buffered Ames medium [44], pH 7.4 maintained at 34–36°C. The major electrolytes in Ames medium are: 120 mM NaCl, 23 mM NaHCO_3_, 3.1 mM KCl, 1.15 mM CaCl_2_, and 1.24 mM MgCl_2_.

### Electrophysiology and light stimulation

Patch electrodes were pulled from borosilicate glass to have a final resistance of 4–8 MΩ. For extracellular loose-patch recording, the electrodes were filled with Ames medium. For intracellular recording the electrodes were filled with the following electrolytes: 110 mM K-methylsulfonate, 10 mM NaCl, 5 mM Na-HEPES, 1 mM K-EGTA, 1 mM Na-ATP, and 0.1 mM Na-GTP. For multi-photon imaging experiments 50–100 µM of Alexa Fluor 488 hydrazide (Invitrogen Corporation, USA) was included in the pipette solution. The liquid junction potential of 10 mV was subtracted from all voltages during analysis. The retina was visualized with infrared differential contrast optics, and ganglion cells with a medium soma diameter and a crescent-shaped nucleus were targeted as potential DSGCs [20]. An extracellular electrode was applied to the soma under visual control through a hole in the inner-limiting-membrane above the cell of interest, and a loose patch recording was formed. After establishing that the ganglion cell was a DSGC and determining its preferred direction (see below), the extracellular recording electrode was removed and an intracellular patch-electrode applied to the same cell for whole-cell recording. During whole cell recordings voltage signals were filtered at 2–4 kHz through the 4-pole Bessel filter of the EPC10 Double patch clamp amplifier (HEKA Electronics Incorporated), and digitized at 20–50kHz. To minimize series resistance errors during whole-cell current-clamp recordings, 10ms hyperpolarizing current pulses were applied and the bridge was balanced to eliminate any instantaneous voltage offsets. Bridge balance was monitored periodically during the recordings.

Light stimuli, generated on a CRT computer monitor (refresh rate, 60 Hz), were focused onto the photoreceptor outer segments through a 40× (NA 0.8) Zeiss water-immersion objective. The percent stimulus contrast was defined as C = 100 * (L-L_mean_)/L_mean_, where L is the stimulus intensity and *L_mean_* is the background intensity. C was set between 30 and 100%. The standard moving stimulus comprised a light or dark bar, moving along its long axis at 800–1200 µm/s on the retina. All light stimuli were centered with respect to the tip of the recording electrode, and thus also with the soma of the ganglion cell. The bar's width was 250 µm, and its length was set to achieve a 1–2 second separation of the leading- and trailing-edge responses. The stimulus area was limited by the aperture of the microscope objective, and covered a circular region 0.5 mm in diameter, which reduced the antagonistic effect evoked by stimulating the surround. The leading edge of the stimulus bar commenced at one edge of the stimulus area and moved until the trailing edge reached the opposite edge. Thus, both leading and trailing edges of the stimulus traversed the whole receptive field of the recorded cell, which evoked both the On- and Off-responses of the DSGC.

### Multi-photon microscopy

A Zeiss Axioskop 2 FS mot equipped with a LSM 510 meta NLO scanhead and a mode-locked Ti/Sapphire laser (Chameleon; Coherent, USA) was used to capture images of DSGC morphology. After break-in, Alexa Fluor 488 hydrazide (Invitrogen Corporation, USA) included in the recording pipette diffused rapidly into the dendritic tree. In some cases the recording electrode was removed from the cell body after the cell had filled with dye before imaging took place. The dye was excited using mode-locked laser light from the chameleon laser tuned to 800–920 nm, and emitted light was collected through the objective, filtered through a BG 39 filter, and detected and digitized with the Zeiss LSM 510 system.

### Digitization and construction of compartmental model

To aid with digitizing stacks of images of tracer-injected cell morphologies, we wrote additional software routines called from the “Image-J” image processing software package. Using Image-J the operator manually traced the cell's dendritic segments and branching pattern, measuring diameters with the caliper tool. Our software saved the morphologies as a collection of nodes and cables. The morphologies were then imported into the Neuron-C simulator [45], [46], and endowed with voltage-gated channels (see “Channel densities” below). Semi-random arrays of presynaptic cells (see below) were constructed automatically by the simulator with a regularity (mean/SD) of 6–10, and synapses were connected between the presynaptic cells and the closest dendrite of the DSGC if it was within a threshold distance (typically 10 um). We set the compartment size small enough (0.03 lambda or less) so that each synapse from a presynaptic array of cells was connected to a different compartment, preserving spatial accuracy. Five morphologies were digitized from confocal stacks and studied along with another more detailed morphology (“ds1e”), which had been traced with a Neurolucida system (Microbrightfield, Inc). Two morphologies explored in detail here, “DS060825” and “ds1e” had ∼750 and ∼3000 compartments respectively. The simulations were run on an array of 15 computers each with 2 or 4 AMD Opteron cores for a total of 48 CPUs, allowing simulations with 50 parameter sets to be run in 24–48 hours.

We performed several types of simulations: calibration, receptive field mapping, single flashed spot, and moving bar. In calibration simulations, we injected various levels of current into the soma and measured the spiking response. Each simulation took ∼1 hour of computing time, and 20–50 simulations were typically run in parallel. In the mapping simulations, we chose a set of points (nodes) in the dendritic tree, and for each point , a protocol measured the conductance threshold (see below). These simulations took roughly 30 minutes per dendritic node, and a sample of several hundred nodes was required for an accurate map of dendritic properties. In single spot simulations, a small spot of synaptic input was turned on over a portion of the dendritic tree and the postsynaptic and soma voltages were recorded. The length of these simulations depended on the spot duration but typically took less than 30 minutes. In the moving bar experiments (see below), we ran 8 simulations in parallel for each of the 8 directions of motion (360°/8 = 45° increments), each of which took ∼45 minutes of computing time. We tested variations in many parameters, including morphology, synaptic input parameters, and channel density parameters, which multiplied the number of necessary simulations, for a total of ∼200,000 simulations to produce the results in this paper.

### Measurement of dendritic attenuation

We measured the attenuation from a dendritic point to the soma by stimulating the point with a low-conductance synapse (200 pS), and computing the voltage attenuation as the ratio of the dendritic and somatic PSP amplitudes [25]. An attenuation less than 1 indicated a dendritic PSP smaller than the somatic PSP. We also computed “synaptic transfer”, a measure of attenuation less sensitive to dendritic R_in_, as the ratio of the PSP amplitudes independently evoked by a dendritic synapse and by a somatic synapse. We performed this measurement over the extent of the dendritic tree by testing many points in independent simulations, producing maps of the dendritic attenuation and input resistance properties (not illustrated).

### Measurement of electrotonic isolation in model dendrites

We computed an approximation to the steady-state space constant (λ_est_) for various points in the dendritic tree to estimate a dendritic region's capability of independently integrating synaptic input. λ_est_ was computed between two points i and j in the dendritic tree by re-arranging the formula for steady-state voltage decay in a passive infinite cable to give:
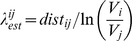
where dist_ij_ is the distance between points i and j. A single synapse was turned on for 100ms to stimulate point i. The simulations were performed using an active model that included Na, K_dr_, K_A_, Ca, K_Ca_, and I_h_ channels, and with a synaptic conductance (50 pS) which always produced a sub-threshold PSP. The quantity 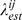 was computed from the steady-state voltages of points within 20–60 um of the site of stimulation and then averaged to give 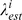. This method thus estimated the space constant based on the local dendritic structure under realistic conditions.

### Measurement of dendritic conductance threshold for spiking

While exploring the dendritic Na^+^ channel density necessary to generate dendritic spikes in response to synaptic input, we found that some regions were more excitable than others, i.e. they produced more spikes. In order to quantify a region's “excitability”, we measured the efficacy of a single synapse to elicit a dendritic spike. The synapse had an exponential release function with a time constant of decay that was longer than the extent of the experiment, and remained “on” for 100ms unless a spike occurred. For a given point in the dendritic tree, we determined the “conductance threshold” (G_thresh_) as the minimum synaptic conductance necessary to elicit a dendritic spike, using an automatic binary search algorithm. This algorithm was run independently on a set of points selected uniformly from the dendritic tree. For each point the algorithm started after the model had equilibrated at a steady-state resting potential, and the model's equilibrated state (voltage of each compartment, synapse states, and channel states) was saved for later use. The initial conductance of the synapse was set halfway between the range of 100pS and 5nS (∼2.5nS). If a spike occurred within a short interval (25–100 ms), the conductance was set to the midpoint of the lower conductance range (100pS to 2.5nS), but if no spike occurred, the conductance was set to the midpoint of the higher conductance range (2.5nS to 5nS). The model was then reset to its original equilibrated state from the saved file and the process was repeated with the new conductance value in the reduced conductance range. The algorithm determined G_thresh_ with an accuracy of 100pS in 7–8 iterations, sufficient to discern the large relative differences in G_thresh_ between distal and proximal regions. Because the model was noiseless, there was no uncertainty in the measurement of G_thresh_.

Although almost all points tested initiated dendritic spikes, the points differed in their ability to successfully propagate spikes to the soma and initiate a somatic spike. To quantify the success of dendritic spike propagation, we injected a synaptic input with the threshold conductance at each dendritic location in independent simulations, recorded dendritic and somatic voltages, and divided the number of somatic spikes by the number dendritic spikes, calling this “propagation efficiency”. The value of propagation efficiency ranged from 0 to 1, with 1 indicating that each dendritic spike successfully propagated to the soma and initiated a somatic spike.

### Morphology of model cells

For the purpose of defining biophysical properties, the morphology of each model was partitioned into 5 regions: dendrites, soma, hillock, thin segment, and axon [28]–[30]. The On-Off DSGC has a bistratified dendritic tree separated into On and Off layers, and each morphology had 3–4 dendritic systems which arose from primary dendrites at the soma. We found each dendritic system was spatially separate, and some arborized in both the On and the Off layers [19]. Dendrites in the Off layer were on average more distant radially from the soma than those in the On layer.

The exact diameter of each dendritic segment that results from digitizing a tracer-injected image is difficult to establish, although the relative diameters between segments can be established with more certainty [21], [47]. Because the diameter of a dendritic segment determines its surface area, capacitance, and axial resistance, we explored the effects of deviations from the digitized morphology. The diameter of each dendritic segment was bracketed by scaling by 0.5 and 1.5, which linearly scaled the dendrites' capacitance and quadratically scaled their axial resistance, affecting the spatial spread of current from the soma [28], [29], [48]. This in turn affected the electrotonic separation between the soma and terminal dendrites, as well as the charging curve, i.e. the voltage trajectory up to the first spike, and time-to-first spike during current injection. Our results were qualitatively similar for models with scaled dendritic diameters. It should also be noted that control simulations where the synaptic inputs were switched to obtain the opposite preferred direction, showed qualitatively similar results, i.e. the synaptic mechanisms could be configured to produce DS in any arbitrary direction for a given morphology.

### Channel kinetics and densities

We set the channel density for each morphological region with fast inactivating Na, Kdr, transient K_A_, high-threshold Ca_L_, I_h_, and K_Ca_ channels similar to previous models [28], [30], [48], [49] (Table 1). We set reversal potentials for Na^+^ at +65mV [50] and for K^+^ at −100mV, which approximated Goldman-Hodgkin-Katz (GHK) potentials calculated for the channel permeabilities assumed in the simulation from the internal recording electrode solution and the external Ames medium [44], [51]. The somatic and dendritic Na^+^ channel densities were encapsulated by two separate parameters. We calibrated these parameters against phase plots from physiological data from rabbit. In order to allow dendritic spikes to initiate and propagate to the soma, the Na-channel density on the dendrites was increased beyond that necessary to produce somatic spike back-propagation into the dendrites [26]–[30]. To generate realistic peak values of dV/dt during the rising phase of a somatic spike we reduced the Na^+^ channel density in the soma and hillock while preserving relatively high dendritic Na^+^ channel densities. To match the physiological data, we slightly altered channel parameters such as the activation and inactivation offsets, and rate multipliers (see below). The channel kinetics were normalized in the simulator software to 22°C, and we took a Q10 value of 2.3 for Na^+^ channel activation as an overall temperature coefficient to match channel kinetics at 35°C [48].

**Table 1:** Values of the standard set of biophysical parameters for regions of the DSGC.

Parameter	Dendrites	Soma	Hillock	Thin Segment	Axon
Na_v_1.2	0	0	0	0	50
Na_v_1.6	35	4	4	100	0
K_dr_	15	15	15	20	10
K_A_	35	35	35	0	0
K_Ih_	0	0.09	0	0	0
sK_Ca1_	0.125	0.125	0.125	0	0
sK_Ca2_	0.05	0.05	0.05	0	0
Ca	0.0140	0.0140	0.0140	0	0
V_rev_	−0.075	−0.100	−0.100	−0.100	−0.100
R_m_	35000	10000	10000	10000	10000

The table shows the parameters for the DSGC models that produced matching F/I and phase plots for 5 different morphologies. In the leftmost column, Na_v_1.2, Na_v_1.6, K_dr_, K_A_, sK_Ca1_, sK_Ca2_, Ca are channel densities, given in mS/cm^2^. V_rev_ is the leak reversal potential (volts), and was treated as a constrained variable when calibrating spike initiation. R_m_ is membrane (leak) resistance in Ωcm^2^. For all morphologies except where specified otherwise, Ri = 200 Ωcm.

### Sodium channel type: Na_v_1.2 vs. Na_v_1.6

Recent immunocytochemical evidence shows that Na_v_1.2 channels are initially expressed at the thin segment during early development but later replaced by Na_v_1.6 channels [52]. Retinal ganglion cells in Na_v_1.6-null mice exhibit impaired (lower) firing rates, and apparently compensate for the missing channel type by increasing the density of Na_v_1.2 channels [53]. These developmental findings suggested that Na_V_1.6 channels play the dominant role in spike generation. Na_V_1.6 channels are known to generate a higher persistent current following a spike, leading to a faster recovery from after-hyperpolarization (AHP), which might be responsible for the shorter inter-spike interval observed in wild type Na_v_1.6 mice [54]. Na_v_1.6 channels activate at more hyperpolarized potentials than Na_v_1.2, which could affect spike shape and rate [54], [55]. We explored the differences between Na_v_1.2 and Na_v_1.6 spike trains using preliminary single compartment models containing either Na_V_1.2 and Na_V_1.6 sodium channels. We found that spikes recorded in the DSGC in response to somatic current injection exhibited a similar fast recovery from AHP that we could only match in the model with the inclusion of Na_v_1.6 channels.

To determine the best match using existing models of Na^+^ channel types for the spike shapes measured in the DSGC, we approximated the kinetics of Na_v_1.2 and Na_v_1.6 channels with Markov models [30], [56], [57]. We explored the differences between Na_v_1.2 and Na_v_1.6 spike trains using preliminary single compartment models containing either Na_V_1.2 or Na_V_1.6 sodium channels.

Both models started with identical K channel densities and kinetics, but one contained Na_v_1.2 channels, and the other Na_v_1.6 channels. To simplify initial calibration of the model, we started with an existing Markov model of Na_v_1.2 Na-channel type and developed it for an approximate match with the real cell's spiking properties, then set the Na_V_1.6 model with similar parameters. We then applied a constant current input to the 2 models and adjusted the densities and kinetics of the Na_V_1.6 channels to produce the best match by eye for spike amplitude, after-hyperpolarization, frequency, and phase plot. In this process we found that the Na_V_1.6 type at any particular voltage was more activated and therefore exhibited a larger open probability. To produce comparable spike amplitude and frequency, we gave the Na_V_1.6 channel rate function an offset of 10mV depolarized from the original Markov activation rate function [57], and to produce comparable spike shapes, we set the Na_V_1.6 density 2–3 times lower than the Na_V_1.2 density. We then took this set of parameters as the initial basis for the spiking properties of our multi-compartment model of the DSGC, and further modified them during the process of Calibration.

### Non-uniform channel densities

Because we found the distal regions of the DSGC to be more excitable, we tested the effects of a higher proximal Na^+^ channel density on dendritic spike propagation and the spatial distribution of dendritic excitability. Recent evidence suggests that some retinal ganglion cell dendrites have a high proximal Na^+^ channel density, although it is not known whether these cells are DSGCs [58]. Previous modeling studies suggest that dendritic Na-channels are necessary for normal spiking [28]–[30], so we set Na-channel density as a gradient where Na^+^ channel density was high in the proximal regions (g_Na1.6_ = 45mS/cm^2^) and declined linearly as a function of integrated cable distance from the soma to a baseline value (g_Na1.6_ = 20mS/cm^2^) for the most distal dendrites, and explored the effect of this gradient on dendritic spiking. We ensured that the minimum density of the most distal dendrites was still high enough to allow initiation and propagation of dendritic spikes (propagation efficiency ∼1), as well as backpropagation of somatic spikes [26].

In a series of initial simulations, we explored the electrotonic properties of the dendritic tree. We found that as the distance from the soma to a dendritic locus increased, the input resistance increased, and the amplitude of a somatic PSP evoked from a constant-strength synapse decreased [22], [33], [59]. This raised the question of whether a compensatory mechanism could modulate the PSP amplitude in the dendritic tips. Because there was evidence for I_h_ currents in the recordings from the real DSGC, we tested its effect in the dendrites. In other neural systems, an I_h_ channel gradient with increasing density and decreasing activation offset with distance from the soma can reduce such a tendency by dampening excitability in distal dendrites [60]. To study the effects of a non-uniform distribution of I_h_ on dendritic excitability, we ran some simulations with dendritic I_h_ channel densities that started at a baseline value close to the soma (g_Ih_ = 0.001mS/cm^2^) and increased linearly with distance from soma to roughly 10 times the baseline value (g_Ih_ = 0.01mS/cm^2^). In those simulations, in order to prevent over-excitability from increased I_h_ in the distal regions of the dendrites, we ramped the activation voltage of I_h_ channels down with distance to 10mV more hyperpolarized in the distal regions than in proximal regions [60].

### Synaptic input

In most simulations, we included synaptic inputs from bipolar and small-field inhibitory amacrine cells. The presynaptic cells were modeled as passive single compartments controlled by a voltage clamp directly set by the stimulus. Each presynaptic cell compartment provided one synapse onto a dendritic compartment of the DSGC. The stimulus for the presynaptic inhibitory amacrine cells was typically spatially offset to simulate the amacrine cells' spatially offset inhibition. The presynaptic voltage passed through threshold and exponential release functions, and the resulting neurotransmitter release was low-pass filtered (tau = 2ms, [30], [45], [46]). To implement noisy vesicle release, the level of released transmitter controlled a nonstationary Poisson (random) release function. The filtered transmitter then passed to a postsynaptic ligand-gated channel, modeled as a Markov 7-state AMPA receptor [61], or a Markov 5-state GABA_A_ receptor [62]. Binding of transmitter to these receptors produced a postsynaptic conductance, whose maximum value was set for each simulation, and ranged from 50pS to 5nS. The reversal potentials for excitatory and inhibitory channels were 0 mV and −68 mV, respectively. Although bipolar and amacrine cells presynaptic to ganglion cells typically make several synaptic contacts [63]–[65], we included only 1 synapse per presynaptic cell for simplicity. This was equivalent to several synapses each with a proportionately smaller conductance within the local dendritic region.

To simulate light stimulation over a receptive field, while avoiding the complications of photo-transduction, light responses of each bipolar and amacrine cell were generated via a “transduction element” which transformed a light intensity in space I(x, y) to a voltage-clamped potential. For example, for DS060825, ∼220 excitatory cells and 180 inhibitory cells were randomly distributed across the On or Off layer and synapically connected to the DSGC's dendritic field. Each transduction element that connected to a cell was assigned a location in space that corresponded to the soma of that cell. Excitation and inhibition were controlled by independently-modulated light stimuli mapped to the same dendritic field. Standard conductance values used except where noted otherwise were excitatory, 80 pS/synapse, inhibitory, 95 pS/synapse. Spatially leading or trailing inhibition was simulated by delaying the onset of the excitatory or inhibitory light stimulus, respectively. For a stimulus moving at velocity v_bar_, delaying the onset of excitatory or inhibitory stimuli by a time Δt produced a spatial offset of Δx = v_bar_/Δt.

### Calibration

**Figure 16:**
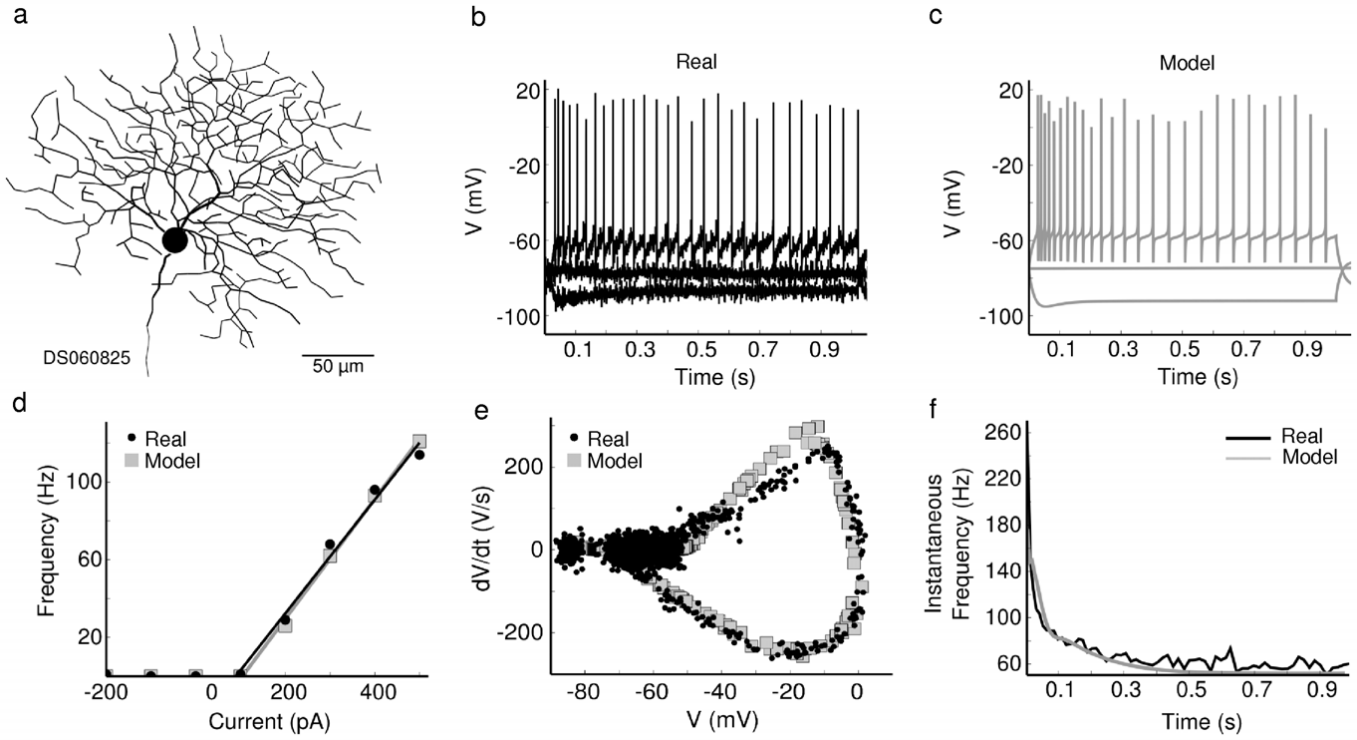
Calibration of the model. Comparison of the results in a real DSGC (DS060825, black) with those obtained from the model (gray). (**a**) Morphology digitized from confocal images of the tracer injected DSGC. The simulation contained ∼750 compartments. (**b**) Somatic voltage recording with current injected at constant current steps of −200pA, 0, and 200pA for DS060825, and (**c**) for the model. (**d**) Spike frequency versus injected current for DS060825 (slope = 0.29Hz/pA, black), and the model (slope = 0.31 Hz/pA, gray). (**e**) Phase plot for somatic spikes elicited by a 300pA current step in DS060825 and the model. (**f**) Instantaneous frequency (inverse of inter-spike interval) plotted against time for real cell (black) and model (gray).

The biophysical parameters provided for each morphology were calibrated to match the F/I curve, spike shape (via phase plot), and ISI curve produced by current injection recordings in the cell from which it was digitized (Figure 16). This produced channel kinetics and densities mentioned in the “Channels” subsection (see above) similar to previously published models of retinal ganglion cells [28]–[30], [48], [49]. In total, five morphologies were modeled. The channel densities thus obtained were closely constrained, because the dendritic Na- and K-channel density is inversely related to the slope of the firing rate vs. input current function [28], [48]. The reason is that if the dendritic channel densities are low, there can be no local dendritic spike initiation, which causes the charge from one spike to surge into the dendrites and return quickly to the soma to initiate another spike too soon. With active back-propagation of spikes into the dendritic tree, the membrane gets charged by the spike and then discharged by K-channels, so any extra charge is prevented from propagating to the soma [28].

Ion channel densities and kinetics were calibrated to electrophysiological and pharmacological data. When I_h_ channels were blocked by application of ZD7288, the DSGC hyperpolarized to 10–20mV below resting potential (data not shown). To simulate this effect, we set the reversal potential of the leak conductance to −100mV, distributed I_h_ channels across the soma and dendrites, and configured them and the other channel types to produce a steady-state resting potential ranging from −70 to −80 mV. Dendritic leak reversal potential was set to assist in calibrating the spike rate, which is particularly sensitive to dendritic channel activation in ganglion cells because their Na and K channels are relatively inactive during the inter-spike interval [28], [48], [66]. We calibrated voltage offsets and densities for Na_v_1.6 and K_dr_ channels by matching phase plots of spikes from physiological recordings (Figure 16e). The Na_v_1.6 and K_dr_ channel activation curves were offset depolarized by 4.5mV and 17mV, respectively. Offsets that significantly varied from these produced mismatched phase plots and a voltage threshold for spiking that differed from the real data.

Calcium channels of both high-voltage-activated (HVA) L-type, and transient low-voltage-activated (LVA) T-type have been found in the soma and dendrites of retinal ganglion cells [67]–[70]. We included L-type Ca^2+^ channels, modeled as a discrete Markov channel with m^3^-like kinetics [71], and we set the Ca^2+^ channel density uniform across the soma and dendrites. We modeled intracellular [Ca^2+^] dynamics in the soma with 10 diffusion shells, each 0.1µm thick, with a Ca^2+^ pump set to give a decay constant of ∼100ms [30]. In the simulations [Ca^2+^]_i_ increased linearly with spike rate, as has been directly observed in DSGC dendrites [3].

In many types of ganglion cells, Ca^2+^-activated K channels (K_Ca_) reduce the firing rate during a prolonged current injection [72]. We included two types of K_Ca_ channels, a small conductance, high [Ca^2+^] affinity, voltage-independent sK_Ca_ channel with an activation time constant near 100ms [73], and another sK_Ca_ channel with a higher [Ca^2+^] affinity and activation time constant near 300ms [74]. K_Ca_ channel densities were distributed uniformly across the soma and dendrites, set to match the cell's frequency-current and ISI curves produced by spike trains at various levels of current injection (Figure 16d–f). The calcium system (Ca^2+^ channels, pump, and K_Ca_ channels) was configured such that [Ca^2+^]_i_ never exceeded 1 µM during repetitive spiking [30]. Both K_Ca_ channel types were active during the inter-spike interval but did not significantly affect spike shape.

### Pre- and postsynaptic DS mechanism

In some simulations, we added excitatory and inhibitory synaptic input from a moving bar stimulus (v_bar_ = 1000µm/s), calibrated to evoke a response similar to physiological data (see above, and Figures 6, 11a,b). We adjusted the spatial separation of the excitatory and inhibitory stimulus components (see “Synaptic Input”) and their corresponding synaptic conductances to approximate the wave shapes of currents (V_hold_ = −75 and 0 mV) recorded in a typical DSGC. We set the rise time for postsynaptic potentials (PSPs) to ∼1 ms and the time constant of decay for EPSCs and IPSCs to 50ms.

For simulations of bar sweeps in 8 directions, we modeled the presynaptic mechanism with overlapping excitatory and inhibitory synapses. The synaptic strength per synapse for excitation (g_e_) and inhibition (g_i_) in each direction *θ* was computed as:
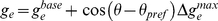


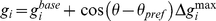
The equations allowed for an arbitrary pref direction to be assigned, typically 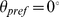. For postsynaptic inhibition, we used a similar equation involving the onset of a temporally delayed inhibition instead of conductance strength:
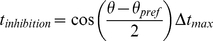
where 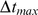 is maximum separation in seconds between the onset of excitation and the onset of inhibition. Given a velocity 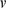, a temporal offset of 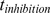 produced a spatial offset of 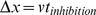.

### Measurement of DS index and directional difference

At a given locus we quantified the direction selectivity of the response by stimulating at evenly-spaced angles distributed over 360 degrees. At each angle the response comprised a vector with length equal to the response amplitude and direction equal to the stimulus direction:
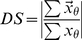
where 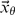 is the spike or peak PSP response vector for a bar swept at angle 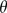, and 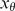 is the magnitude of the response. The vector sum represented the directional response, and its length, normalized by the sum of response amplitudes, represented the “DS index” or DSI, and ranged from 0 to 1 [3], [7]. For comparing PSP and spiking responses, the peak PSP was computed by first digitally removing spikes (“spike-blanking”) [3]. For some tests, we calculated the directional difference between PSPs as the peak amplitude of the preferred direction PSP minus the peak amplitude of the null direction PSP.

### Accuracy of synaptic conductance recorded in DSGC soma

**Figure 17:**
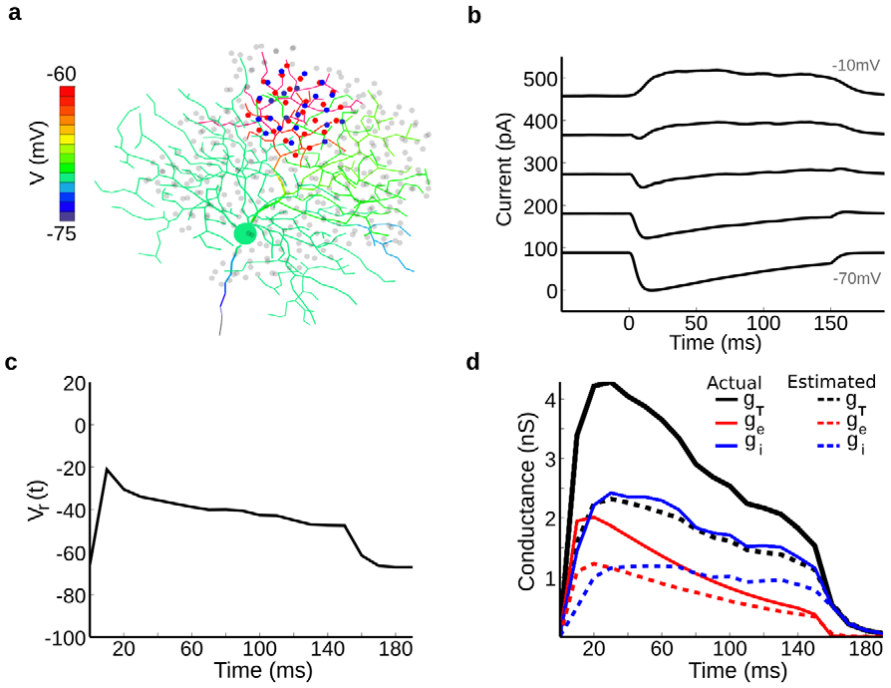
Estimate of the errors in the measurement of the synaptic conductance in a realistic model of a DSGC. We estimated excitatory and inhibitory conductances in our model after Taylor and Vaney [7] and compared them to actual conductances produced by the model. (**a**) Color-map shows voltage throughout the DSGC15 ms after the onset of a spot of synaptic input in a distal region, while the soma was voltage clamped at −70mV. The dendrites are not isopotential with the soma, but are more depolarized by up to10mV. Dots are the presynaptic cells, red = excitatory, blue = inhibitory, gray = not stimulated. (**b**) Somatic current recorded at different holding potentials, starting at −70mV (bottom) and increasing (15mV steps) to −10mV (top). (**c**) The cell zero-current potential V_rev_(t) as a function of time during the synaptic inputs. (**d**) The total conductance (black) comprised the sum of excitatory (red) and inhibitory (blue) conductances. The dotted lines indicate the synaptic conductances estimated by analyzing the somatic voltage-clamp currents in (b), and the solid lines show the actual conductances active in the dendrites. The amplitude errors in the estimated conductances ranged from 50–100%.

In order to determine how postsynaptic inhibition suppresses spikes in the DSGC during null-direction stimulation, we first attempted to determine the magnitude of the inhibitory synaptic conductances as measured from the soma. Excitatory and inhibitory synaptic conductance components are often estimated from the currents recorded at the excitatory and inhibitory reversal potentials (e.g. [17]), or by measuring currents over a range of holding potentials and calculating the excitatory and inhibitory synaptic conductances from the synaptic current-voltage relation [6], . With either approach, incomplete space-clamp inevitably leads to errors in the magnitudes of the conductance estimates [32]. To investigate how estimates of the synaptic conductance derived from recordings at the soma deviate from actual conductances, we simulated synaptic input to the DSGC model and estimated the conductances during somatic voltage clamp (Figure 17).

During a small voltage step, accurately fitting the capacitive transient in the DSGC requires a sum of exponentials, implying that the cell is not isopotential [7]. We stimulated a distal area with a spot of co-localized excitatory and inhibitory input, and verified that the dendrites were not isopotential with the soma (Figure 2a). When the soma was voltage clamped at holding potentials above or below resting potential, current leaked out through the dendrites and distal current flow was impeded by axial resistance, causing a voltage difference in the distal dendrites. At more depolarized holding potentials the model was less isopotential, and the postsynaptic current produced by the spot, computed by summing all individual synaptic currents, was larger than the synaptic currents recorded at the soma. This produced a lower I–V slope at each time point during the synaptic response, which led to a more depolarized synaptic reversal potential estimate (Figure 2c) and an underestimate of the total conductance (Figure 17d, black). For this spot stimulus, the excitatory and inhibitory synaptic conductances were underestimated by factors of ∼40% and ∼50%, respectively (Figure 17d, red, blue). Inhibitory conductances were underestimated more than excitatory conductances because space clamp errors were greater at depolarized clamp potentials, leading to a lower slope on the I/V plot and a reversal potential more positive than expected. A more positive synaptic reversal potential is interpreted as a relatively larger excitatory component or smaller inhibitory component. When the spot of synaptic input was localized over the soma and proximal dendrites, errors in the synaptic conductance estimates were minimal. In a similar model using a moving bar, the synaptic conductances were under-estimated by a similar amount in the distal dendrites and the soma.

## Supporting Information

Video S1 Movie of the simulation of the DSGC, showing dendritic spike initiation, forward propagation to the soma, and back propagation to the remainder of the dendritic tree. Explanatory text is included in the movie. The movie is in H.264 MPEG-4 format.
(7.54 MB MOV)
